# Recent Advances in Batteryless NFC Sensors for Chemical Sensing and Biosensing

**DOI:** 10.3390/bios13080775

**Published:** 2023-07-31

**Authors:** Antonio Lazaro, Ramon Villarino, Marc Lazaro, Nicolau Canellas, Beatriz Prieto-Simon, David Girbau

**Affiliations:** 1 Department of Electronics, Electrics and Automatic Control Engineering, Rovira i Virgili University, 43007 Tarragona, Spain; ramon.villarino@urv.cat (R.V.); marc.lazaro@urv.cat (M.L.); nicolau.canyellas@urv.cat (N.C.); beatriz.prieto-simon@urv.cat (B.P.-S.); david.girbau@urv.cat (D.G.); 2 Catalan Institution for Research and Advanced Studies (ICREA), Pg. Lluís Companys 23, 08010 Barcelona, Spain

**Keywords:** near-field communication (NFC), batteryless, potentiostat, electrochemical sensor, point-of-care, wireless RF energy harvesting, chipless sensor, green electronics

## Abstract

This article reviews the recent advances in the field of batteryless near-field communication (NFC) sensors for chemical sensing and biosensing. The commercial availability of low-cost commercial NFC integrated circuits (ICs) and their massive integration in smartphones, used as readers and cloud interfaces, have aroused great interest in new batteryless NFC sensors. The fact that coil antennas are not importantly affected by the body compared with other wireless sensors based on far-field communications makes this technology suitable for future wearable point-of-care testing (PoCT) devices. This review first compares energy harvesting based on NFC to other energy-harvesting technologies. Next, some practical recommendations for designing and tuning NFC-based tags are described. Power transfer is key because in most cases, the energy harvested has to be stable for several seconds and not contaminated by undesired signals. For this reason, the effect of the dimensions of the coils and the conductivity on the wireless power transfer is thoroughly discussed. In the last part of the review, the state of the art in NFC-based chemical and biosensors is presented. NFC-based tags (or sensor tags) are mainly based on commercial or custom NFC ICs, which are used to harvest the energy from the RF field generated by the smartphone to power the electronics. Low-consumption colorimeters and potentiostats can be integrated into these NFC tags, opening the door to the integration of chemical sensors and biosensors, which can be harvested and read from a smartphone. The smartphone is also used to upload the acquired information to the cloud to facilitate the internet of medical things (IoMT) paradigm. Finally, several chipless sensors recently proposed in the literature as a low-cost alternative for chemical applications are discussed.

## 1. Introduction

In modern societies, there is a progressive aging of the population and an increase in people with chronic diseases that require continuous monitoring (e.g., diabetes) [1]. In this context, home health care becomes more relevant every year, underpinned by the growing number of dependent people and people with mobility restrictions, as well as the increased life expectancy associated with certain chronic processes. In addition, the COVID-19 pandemic has accelerated the need to implement telemedicine services due to the saturation of primary care centers and hospitals [2].

The internet of medical things (IoMT) paradigm refers to the network of medical devices, applications, and systems that are interconnected through the internet and work collaboratively to collect, transmit, analyze, and act on health-related data. IoMT is a subset of the broader internet of things (IoT) concept, specifically focused on healthcare and medical applications. Here, the concept of point-of-care testing (PoCT) arises, defined as the medical diagnostic testing at or near the point of care, in contrast to the historical pattern in which testing was limited entirely or primarily to medical settings [3,4,5]. In addition, testing devices are important in developing countries to guarantee adequate medical diagnosis in remote areas. The development of this concept in the frame of remote monitoring at the home of specific biomarkers by using sensors and communication technologies opens new opportunities and a new paradigm in the digital transition in the field of health. To this end, low-cost wearable devices pave the way for the new generation of PoCT devices [6]. It is in this context that the need for biosensors integrated in wireless devices arises.

**Figure 1:**
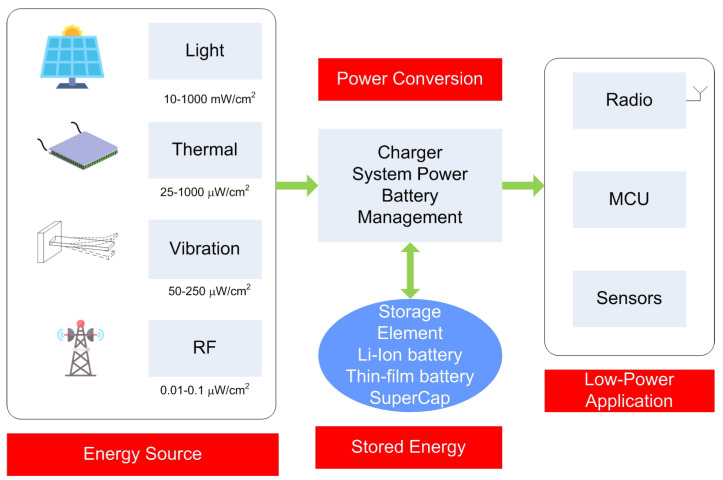
Available energy sources for energy harvesting and block diagram of WSN mote supported with energy harvesting.

There is a growing interest in wireless sensor networks (WSNs) for a wide range of applications. These sensors are used in many cases for collecting data obtained from environments where regular access is not possible or impractical. The use of wireless networks allows rapid deployment over large areas without the need for a major infrastructure. Nonetheless, the most important problem with these systems is that they mostly run on batteries. Due to the limited lifetime of batteries, they must be replaced periodically. The extension of this lifetime can be achieved by increasing the interval between measurements or applying other strategies to reduce the consumption of the nodes, which normally depends on the end application. Even if rechargeable batteries are used, they must be recharged periodically. For this reason, different energy-harvesting methods have been investigated to recharge or increase the lifetime of batteries from different energy sources. Figure 1 shows a typical block diagram of a wireless sensor node with energy harvesting. An energy storage system (either battery or supercapacitor) is required in most applications because, normally, the external power source has limited availability and can only provide sufficient power intermittently. Table 1 shows the limits in power density available from different energy-harvesting sources.

There are small-scale energy sources that generally cannot produce an output power comparable to industrial solar or wind generators. Solar energy is the most efficient natural energy source available for outdoor applications. However, for indoor applications, it is important to note that the efficiency of photovoltaic cells is very low. Typically, the light intensity under artificial lighting conditions in hospitals or offices is less than 10 W/m${}^{2}$ compared to 100–1000 W/m${}^{2}$ in outdoor conditions [7]. For example, monocrystaline solar cells have an efficiency of less than 1–3% under typical indoor lighting condition, although amorphous solar cells have been found to have slightly higher efficiencies of 3–7% under indoor conditions, but they are more expensive than the first [8].

Another source of energy is the thermal source. This kind of energy harvesting is based on a thermoelectric generator (TEG). A TEG module is essentially made up of many PN pairs (doped semiconductors P and N types) placed in series. The Seebeck effect produces a voltage difference across each PN junction when a temperature gradient is created between the two sides of the PN junction. TEGs are often used in situations where there is excess waste heat, such as in industrial processes, automotive applications, or in power generation from sources like biomass or geothermal. TEGs typically have lower efficiency compared to traditional power generation methods like internal combustion engines or steam turbines. Their efficiency is often in the range of 5% to 10%. Thermoelectric generators (TEGs) have the potential to be a valuable technology for wearable applications, where the human body itself can serve as a heat source. Wearable TEGs can be used to harness the body’s natural heat and convert it into electrical energy to power various electronic devices or charge batteries. In addition, they should be compact, lightweight, and flexible to integrate seamlessly into clothing or wearable devices. Flexible thermoelectric materials and thin-film technologies may allow the development of TEGs that conform to the shape of the body and do not hinder movement or comfort. Effective thermal management is crucial to maintain a temperature gradient across the TEG. Adequate insulation on the cold side and optimized heat dissipation on the hot side are essential to maximize the power output. For gradient differences of 5 K, only 60 μW/cm${}^{2}$ is usually obtained. The low-voltage gradient obtained from TEG on the body (about 50 mV) requires a specialized ultralow voltage step-up converter (e.g., LTC3108, from Analog Devices, Wilmington, NC, USA) to charge a storage supercapacitor.

Vibration microgenerators, also known as vibration energy harvesters or vibration-powered microgenerators, are a type of small-scale energy-harvesting device that convert mechanical vibrations or movements into usable electrical energy. These microgenerators are designed to scavenge ambient vibrations from the environment, such as human motion, machinery vibrations, or natural vibrations, and transform them into electrical power for various applications. Most vibration microgenerators use piezoelectric materials that generate an electric charge when subjected to mechanical stress or vibrations [9].

Energy harvested from RF sources is widely available in both urban and semi-urban areas, and therefore constitutes a promising candidate for ambient energy-harvesting sources [10]. There is an increasing number of different electromagnetic power sources, such as cellular mobile base stations, digital television (TV) towers, and Wi-Fi routers that use relatively high-power transmitters that can be used as power sources. A rectenna often based on rectifying Schottky diodes, performs the conversion from the RF signal captured by an antenna to DC. The rectenna efficiency depends on the input RF power and consequently on the distance to the RF source (base station). Therefore, the rectenna under indoor environments receives very small input RF power densities due to the attenuation of walls and multipath effects.

One special case of WSN is wearable and point-of-care testing devices. Usually, these devices are powered by batteries. Lithium-ion rechargeable batteries are preferred for wearable devices, while dry cells continue to be the preferred choice for other devices. For simple devices, the extra cost of the charging components and battery monitoring significantly increases their final price. In the age of smartphones, the data acquired by these sensors are collected wirelessly via Bluetooth (e.g., Bluetooth low-energy), Wi-Fi, or NFC, are often displayed on the mobile screen via a bespoke app, and, in some cases, can be uploaded to the cloud. Moreover, the use of the smartphone increases the user friendliness of the system. Battery life is an important consideration for users when choosing between similar devices on the market. In many cases, users stop using the devices when their batteries require frequent recharging. This poses an environmental risk due to the presence of toxic elements in this growing generation of devices which are often not properly disposed of. Unfortunately, proper recycling is often hampered by the cost or difficulty of separating these toxic components from electronic devices. These toxic components may also pose a health risk if they reach aquifers, rivers, and seas [11]. Therefore, it is necessary to progressively consider green electronics concepts, including biodegradable materials in the new designs of wearable and smart devices. In this direction, the first step is the use, when possible, of devices without batteries. At this point, the difference between wearables for indoor applications and WSNs that are mostly used outdoors (e.g., agriculture or civil infrastructure monitoring) must be highlighted due to the limited energy availability from harvesting energy sources or the area or mass required to collect the energy. With the exception of heel-strike harvesting in electric shoes or solar cells in bright light, available powers generally hover at mW or μW levels (see Table 1) [12].

One interesting solution for some specific applications is the use of passive RFID (radio frequency identification) tags, which can include sensors. The energy to power the tag is obtained from the electromagnetic signal generated by the reader used to interrogate it, instead of using electromagnetic signals present in the environment, coming from other systems. The presence of the reader ensures the power source, but the amount of power that can be extracted is limited and depends on the distance to the reader and the type of RFID. Communication in RFID systems is performed by backscatter (load modulation of an antenna) [13,14]. Therefore, wireless communication is carried out efficiently without the need for high-power transmitters. Passive RFID systems in the UHF band [15,16] have experienced great growth, especially for logistics and identification applications. Although they are less widespread, there are UHF-integrated circuits that include AD converters, allowing for the implementation of sensors (e.g., wireless temperature sensors) [17,18]. Currently, the sensitivity of commercial UHF RFID integrated circuits is on the order of −21 dBm with ranges up to 9–10 m, depending on the reader antenna used. In the case of UHF tags that include sensors, this sensitivity is reduced to −12.5 dBm to 0 dBm due to the higher energy required to power the internal electronics of the integrated circuit, which is more complex [19]. Therefore, the reading range of these sensor tags is reduced to distances of the order of 1 m [20]. UHF tag antennas (e.g., inlays) are detuned, and their losses increase in the presence of high-permittivity materials (e.g., the human body or liquids) [20] and metals [21]. Another problem associated with RFID technology is related to the different national regulations, which affect parameters such as the frequency band [14]. This makes it difficult to design antennas that provide a uniform read range for all regulations; different designs of broadband or dual-band antennas for RFID tags have been proposed [22]. As a result, special ad hoc designs are required for these environments, increasing the cost and size of the tag.

Instead of RFID systems based on far-field communication, there are RFID systems based on near-field communications (NFC) [23]. The operating principle of NFC is the inductive coupling between the reader and tag coil antennas [24], and the most popular is the one which works at 13.56 MHz. Although HF-based RFID tags have been used for many years, the take-off of this technology began in 2004 with the creation of the NFC forum [25] by Nokia, Sony and Philips, and other semiconductor companies. The forum enforces strict standards that manufacturers must adhere to when using their NFC-compatible devices. This ensures that NFC is secure and remains easy to use with different versions of the technology. Compatibility has been key to the growth of NFC as a popular payment and data communication method. In 2010, the first Android smartphone with NFC reader was introduced in the market. Since then, NFC technology has not stopped growing. Finally, the COVID-19 pandemic has made NFC payment more widespread, thanks to its reduced contact [26].

Apart from payment applications [27,28] and smart posters [29,30], in recent years, NFC technology has aroused great interest in the field of batteryless sensors [31]. The main NFC manufacturers have introduced in the market NFC-integrated circuits with energy-harvesting capabilities (e.g., M24LR, ST25DV from ST Microelectronics, or NT3H11 and NTAG 5 from NXP). These NFC ICs have a voltage output that can power external devices, such as sensors and microcontrollers. A few mW (<16 mW) can be drawn by placing the tag a few cm away from the reader, depending on the size of the antenna used [32,33]. This power is enough to feed sensors, covering a wide range of applications, and is considerably higher than that which can be harvested from UHF RFID devices or other indoor power sources in wearable devices. Unlike UHF RFID technology, which is difficult to integrate into smartphones due to the need for expensive, bulky, dedicated, high-consumption readers to generate enough energy to power the tags in the far-field region, NFC readers are included in most smartphones and even in smartwatches. Therefore, no additional readers are required for these new battery-free NFC-based sensors. Their standardization allows rapid application development, as data can be stored in the internal memory of the NFC IC and read using the tools provided in the smartphone application programming interfaces (APIs). Another advantage, as described below, is the relatively simple design of NFC antennas, which have robust electrical behavior on materials with high dielectric permittivity and can be used in on-body and implanted medical devices (IMDs) [34], in contrast with other communication systems that work at higher frequencies in the far field (e.g., UFH RFID or Bluetooth antennas). Batteryless sensors are eco-friendly, but they are not suitable for data-logging applications because they can only perform their sensing functions when the reader is close to them. To overcome this intrinsic limitation, a batteryless sensor can be converted into a simple data logger if a battery is included, but this limits their interest compared to other systems such as Bluetooth low-energy, which can also be read from a smartphone, allowing a longer reading range and a higher data transmission throughput. This last consideration provides batteryless NFC technology with great potential for several sensing applications, particularly for wearable and biomedical ones.

The paper is organized as follows. Section 2 describes the structure of batteryless NFC sensors based on energy harvesting and some guidelines for their design. Section 3 reviews several recent works on NFC sensors available in the literature which focus on biomedical applications. In the discussion Section 4, an intercomparison is made between the different sensor technologies according to their principle of operation. Finally, conclusions and future perspectives are provided in Section 5.

## 2. NFC Sensor Overview and Theoretical Background

### 2.1. System Overview

**Figure 2:**
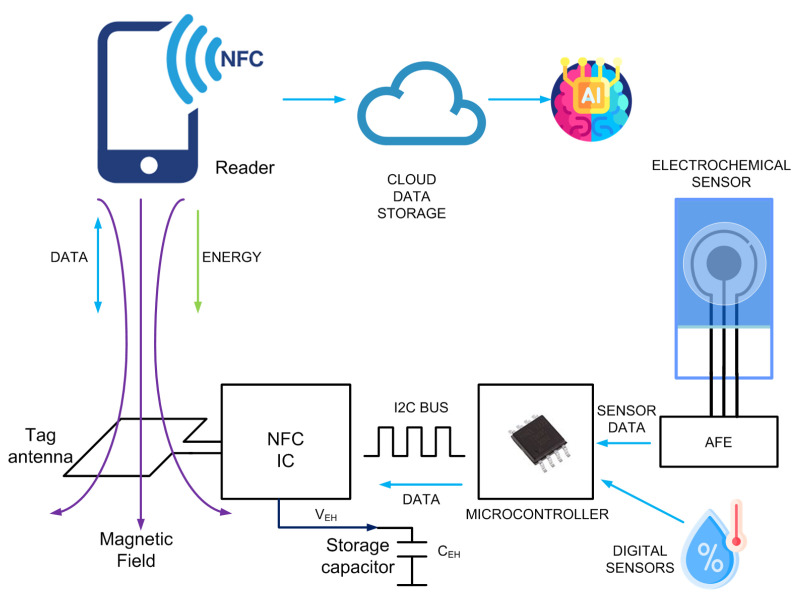
Scheme of a typical block diagram of a batteryless NFC sensor.

Figure 2 shows the typical block diagram of a batteryless NFC sensor. It consists of an NFC IC with a loop antenna, a microcontroller (MCU), an analog front end (AFE) and sensors. The NFC IC performs two tasks: collects the energy from the electromagnetic field generated by the reader to power up the sensor, and establishes data communication between the sensor and the reader.

The main difference between an NFC IC that includes energy harvesting compared to those designed for RFID cards is the presence of an energy-harvesting voltage output. When the magnetic field is high enough, this energy-harvesting voltage output is enabled in the IC configuration registers, and the NFC IC provides a DC voltage capable of powering other circuits. This output is connected to the internal rectifier of the IC, which obtains the energy from the incoming RF field. In most NFC ICs, the output is regulated, and external circuitry can be powered directly without the need for a battery.

**Figure 3:**
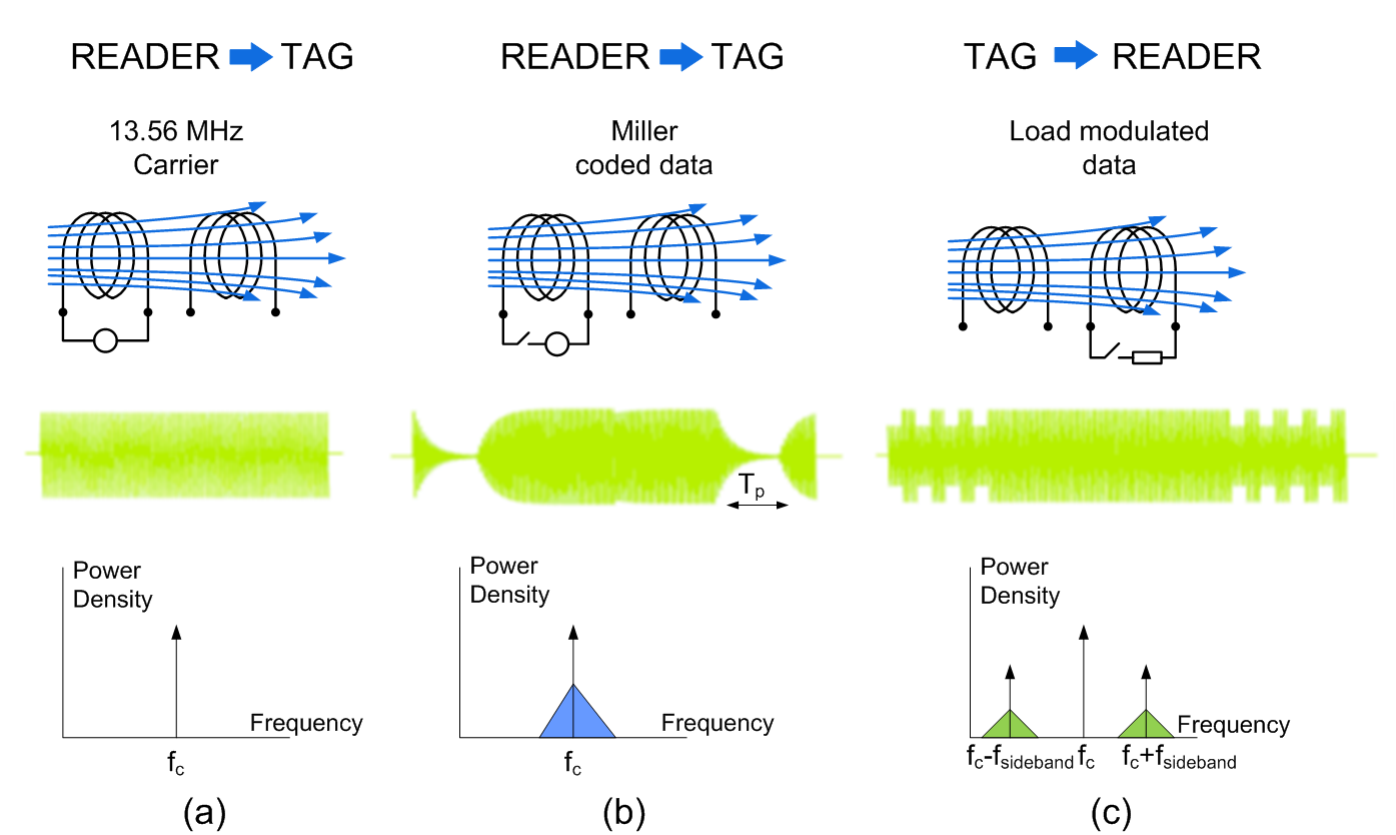
Schema of time-domain waveforms and spectra for the NFC reader and tag communication: (**a**) tag power up (the reader transmits a carrier at 13.56 MHz), (**b**) transmission of reader-to-tag commands by modulation of the carrier, (**c**) transmission of tag-to-reader data by load modulation using a subcarrier.

The reader–tag communication is represented schematically in Figure 3. It is based on inductive coupling between two coil antennas and works at the 13.56 MHz ISM band. The reader starts the communication by generating an unmodulated magnetic field at the carrier frequency $f_{c}$ = 13.56 MHz. Once the NFC tag is in the close vicinity of the reader, inductive coupling occurs between both coils (Faraday’s law of induction), allowing to power the tag. Then, the reader sends commands modulating the carrier using ASK modulation. The tag responds to the command by transferring the data stored in the internal memory through load modulation (switching the tag antenna load using a subcarrier). The data transmitted by the tags based on the ISO 14443 standard modulate a subcarrier whose value is $f_{sideband}=f_{c}$/15 = 847.5 KHz, whereas those based on standard ISO 15693 support one or two subcarriers. In the event of using a single subcarrier, the frequency $f_{sideband1}$ of the subcarrier load modulation is $f_{c}$/32 (423.75 kHz). When two subcarriers are used, the frequencies $f_{sideband1}$ and $f_{sideband2}$ are $f_{c}$/32 (423.75 kHz) and $f_{c}$/28 (484.28 kHz), respectively.

General-purpose NFC ICs do not include an analog-to-digital converter (ADC); for this reason, a microcontroller is needed to acquire the data from sensors. However, for specific applications, there are ICs that incorporate these functions, and the trend of most of them is to use the ISO 15693 standard. However, it is also possible to find devices based on ISO 14443-3. Table 2 summarizes some features of several commercial NFC ICs (this is an updated version of the table presented in [31], which does not consider the models that are now obsolete). The data read by the ADC microcontroller are sent to the EEPROM memory of the NFC IC through a two-wire $I^{2}C$ serial interface and are encoded in a standard NFC data exchange format (NDEF), which is readable using NFC protocol. The microcontrollers used in the design of these batteryless sensors are usually low cost and low power (typically 8-bit) since they do not need to perform very complicated functions or complex calculations, which in most cases can be performed by the reader. The clock speed of the microcontroller may be low (e.g., to 1 MHz) in order to reduce power consumption. As the number of logic gates in 8-bit microcontrollers is less than that in 16-bit ones, their power consumption in active mode is smaller. Modern 8-bit microcontrollers can generally work at voltages down to 1.8 V and therefore can operate at high current load modes that provide low energy-harvesting output voltages. With this configuration, the current consumption of the microcontroller in active mode can be less than 1 mA, and accordingly, it can be powered by the NFC IC.

A storage capacitor $C_{EH}$ is connected to the energy-harvesting output. The value chosen must be the one that avoids voltage drops during the modulation pauses of the reader (Miller modulation type), which are of $T_{p}$ duration. A low dropout regulator can be used to reduce voltage fluctuations during reader interrogation periods. In both ISO 14443-3 and ISO 15693 standards, $T_{p}$ is about 10 μs. The voltage drop in the storage capacitor can be calculated from the following expression:

(1) $$V_{drop}=\frac{I_{L}\cdot T_{p}}{C_{EH}}$$

where $I_{L}$ is the load current obtained from the energy-harvesting output. For a value of $C_{EH}=1\;\mu F$ and load currents of 2 mA, the voltage drop at the input of the regulator is around 20 mV, which is acceptable for low-drop regulators. Note that this dropout is like a small burst that can propagate to the analog front end (AFE), introducing noise in the ADC lines, which can further be amplified for example, in the potentiostat’s high-gain transimpedance amplifier (TIA) when using electrochemical sensors.

### 2.2. Tag Design Considerations

**Figure 4:**
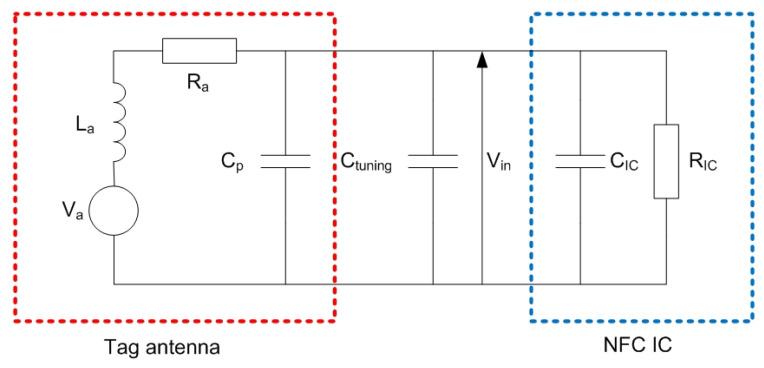
Equivalent circuit of the tag antenna and NFC IC.

Figure 4 shows an equivalent circuit for the NFC tag. It consists of the following parts: the equivalent voltage at the antenna, the equivalent electrical circuit of the tag antenna and the input equivalent circuit of the NFC IC modeled as a parallel RC circuit, and a tuning capacitor $C_{tuning}$. The equivalent electrical circuit of the tag antenna consists of an inductance $L_{a}$ connected in series with resistance $R_{a}$ that models the antenna losses (DC plus AC due to skin effect). The parallel capacitance $C_{p}$ is associated to the parasitic capacitance of the antenna and layout interconnections. The NFC IC input impedance exhibits a non-linear behavior [34]. Whereas the equivalent chip capacitance $C_{IC}$ hardly depends on the input voltage, the same does not happen with the resistance $R_{IC}$, which also depends on the input IC voltage and the load connected to the energy-harvesting output. $R_{IC}$ is on the order of several hundred Ω to kΩ [35,36].

The equivalent voltage at the antenna $V_{a}$ induced by the AC variation of the magnetic flux is obtained from Faraday’s law:

(2) $$V_{a}=j2\pi f\mu_{0}A\cdot N\cdot H_{av}$$

where *f* is the frequency, *A* is the area of the printed coil antenna, *N* is the number of loops of the coil, $\mu_{0}$ is the vacuum magnetic permeability, and $H_{av}$ is the average magnetic field of the tag antenna. For antennas loaded with magnetic material (e.g., ferrite sheets), $\mu_{0}$ must be replaced by the effective magnetic permeability (which is defined as the ratio of the inductances loaded with and without ferrite).

The magnetic field depends on the distance between the tag and the reader and the alignment of the antennas. For a correct operation of the tag, a minimum voltage $V_{min}$ at the input of the IC is required. By analyzing the circuit of Figure 4, the voltage at the input of the chip can be obtained [37], as well as the minimum required magnetic field given by [31,37]

(3) $$H_{min}\approx \frac{\sqrt{{\left(1-{\left(f/f_{r}\right)}^{2}\right)}^{2}+1/Q_{T}^{2}}}{2\pi f\mu_{ef}A\cdot N}\cdot V_{min}$$

where $\mu_{ef}$ is the effective magnetic permeability in the case of considering ferrite materials.

To achieve high energy transfer, the resonance frequency of the tag has to be adjusted to 13.56 MHz (which is the operating frequency of the NFC). The tag’s resonance frequency is given by

(4) $$f_{r}=\frac{1}{2\pi }\sqrt{\frac{R_{IC}+R_{a}}{L_{a}R_{IC}\left(C_{IC}+C_{p}+C_{tuning}\right)}}\approx \frac{1}{2\pi \sqrt{La(C_{IC}+C_{p}+C_{tuning})}}$$

$Q_{T}$ is the total quality factor of the tag at the resonance frequency, which is a combination of the quality factor of the antenna $Q_{a}$ and that of the NFC IC $Q_{L}$:

(5) $$Q_{T}=\frac{1}{1/Q_{a}+1/Q_{L}}=\frac{1}{\frac{R_{a}}{2\pi f_{r}L_{a}}+\frac{2\pi f_{r}L_{a}}{R_{IC}}}\approx \frac{R_{IC}}{2\pi f_{r}L_{a}}$$

For antennas with high-quality factors, the total quality factor can be approximated by that of the NFC IC ($Q_{L}$).

**Figure 5:**
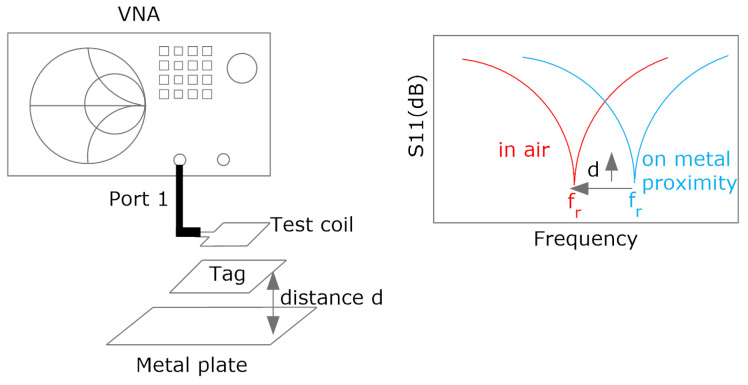
Setup for the measurement of the resonance frequency by means of a VNA, with the presence of a metal plate. Effect of the distance to the metallic object on the measured S_11_.

**Figure 6:**
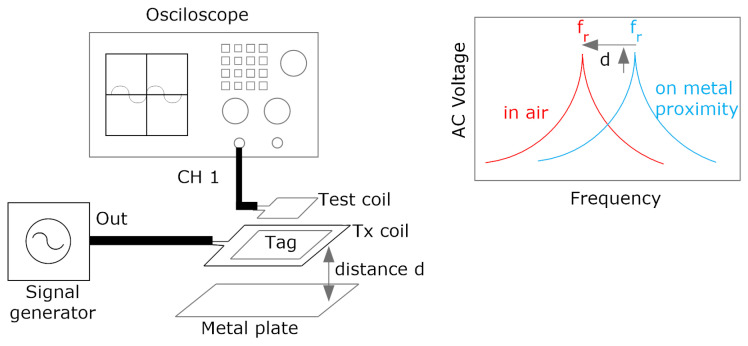
Setup for the measurement of the resonance frequency using a signal generator and an oscilloscope, with the presence of a metal plate. Effect of the distance to the metallic object on the measured voltage.

Some guidelines can be obtained by analyzing (3). Considering the inductance of the designed antenna, the resonance frequency $f_{r}$ has to be tuned to 13.56 MHz to reach the minimum value of $H_{min}$. To this end, the tuning capacitance can be calculated from Equation (4). In this procedure, it is necessary to know the value of the antenna inductance, which can be obtained from simulations or from measurements of antenna impedance performed with a vector network analyzer or an impedance analyzer. It is essential to reduce the detuning of the tag caused by the presence of metal parts, such as the mobile casing, which reduces the inductance due to the image currents induced in the metal [31,38]. Therefore, the measurement must be made under conditions equivalent to those of the operation of the tag. One of the advantages of NFC over other RFID technologies at higher frequencies (e.g., UHF band) is its robustness against the presence of high-permittivity materials such as some liquids or the body itself. As these materials are not magnetic, the effect of the permittivity induces the variation of the parasitic capacitance, which is of the order of few pF, below the typical input IC capacitance (around 30 pF). The final tuning process can be carried out by measuring the S_11_ reflection coefficient with a test coil connected to a VNA (Figure 5). The adjustment should be performed in the presence of a mobile phone or a metal plate at the desired reading range in order to simulate the actual operating conditions. The resonance frequency is obtained from the frequency of the notch of the S_11_. Measurements can be made with low-cost VNAs operating at 13.56 MHz, such as NanoVNA, or with spectrum analyzers equipped with a reflectometer. Alternatively, a setup consisting of a signal generator connected to a transmitting antenna and a receiving antenna connected to an oscilloscope can be used to measure the resonance frequency of the tag [39] (Figure 6); the measurement consists in sweeping the frequencies with the generator in such a way that the resonance frequency is obtained when the maximum of the voltage is measured at the oscilloscope. Simple test coils can be handmade with a wire coil connected to a coaxial connector.

The selection of the antenna depends on several factors, including the final application of the tag. However, some restrictions should be considered. The antenna (including its parasitic elements) and the input impedance of the chip act as a bandpass filter. If the total quality factor of the tag is too high, the sidebands are attenuated, and the communication between the tag and reader becomes worse. The maximum value of the quality factor can be estimated from the bandwidth using the following relationship:

(6) $$Q_{Tmax}=\frac{f_{0}}{BW}\approx \frac{f_{0}}{2f_{sideband}}$$

where $f_{0}$ = 13.56 MHz is the tag operation frequency, and $f_{sideband}$ is the frequency of the modulated subcarrier, which depends on the standard. For tags that comply with ISO 14443 ($f_{sideband}=f_{0}/16$) and ISO 15963 ($f_{sideband}=f_{0}/28$) standards, the maximum total quality factors are 8 and 14, respectively. Note that these quality factors are notably lower than that required in the reader, which is of the order of 40 [31]. This maximum value of the quality factor imposes a minimum inductance value, which can be estimated from Equation (5). For antennas with a high quality factor, the chip quality factor predominates and can be estimated from [40]

(7) $$L_{a}>\frac{R_{IC}}{2f_{0}Q_{Tmax}}$$

For NFC ICs with energy harvesting, the value of $R_{IC}$ is of the order of 525 Ω[41] so that the minimum inductance values are 770 nH and 440 nH for the tags that work under ISO 14443 and ISO 15963 standards, respectively. The maximum value of the antenna inductance is limited by the resonance condition (4), which for an NFC IC with a $C_{IC}$ of 30 pF results in a maximum inductance of 4.5 μH. Low inductance values must be avoided since more current flows through the tag and, as a result, the coupling between the reader and the tag causes a reader impedance mismatch. To increase the energy transfer, the coupling coefficient between the antennas of both the tag and the reader should be as high as possible. To this end, the size of both antennas must be similar but not identical to avoid the detuning effect at short distances [42]. Modern mobiles embed the NFC antenna around the camera aperture or over the battery case, with typical sizes around 2–2.5 cm${}^{2}$ [43,44,45,46].

Square, circular, hexagonal, and octagonal inductors are widely used for the design of NFC antennas. For a particular shape, the inductance is obtained from the number of turns *N*, the width *w*, the space between turns *s*, the outer diameter $D_{out}$ and the inner diameter $D_{in}$. For single-layer loop antenna designs, simple analytical expressions can be used as starting point, and the inductance can be calculated using the modified Wheeler expression [47]:

(8) $$L_{a}=K_{1}\mu_{0}N^{2}\frac{D_{avg}}{1+K_{2}\rho }$$

where $\mu_{0}$ is the vacuum magnetic permeability constant ($4\pi \cdot 10^{-7}$ H/m) and $D_{avg}$ is the average diameter:

(9) $$D_{avg}=\left(D_{out}+D_{in}\right)/2$$

and ρ is the fill factor that represents how hollow the inductor is. It is defined as

(10) $$\rho =\frac{D_{out}-D_{in}}{D_{out}+D_{in}}$$

The coefficients $K_{1}$ and $K_{2}$ are shape dependent and are given in Table 3. Other alternative expressions for the inductance calculation are the current sheet approximation and data-fitted monomial [48].

Typically, the space between turns *s* is chosen in the order of *w*. This value improves the magnetic coupling between strips and reduces the size of the inductor. Large spacing *s* is only considered to reduce the strip capacitances. The accuracy of the Wheeler’s inductance expression is 8% [48]. In cases where other antenna shapes are considered, multilayer designs are intended, or higher accuracy is desired, it is essential to use electromagnetic solvers. The antenna quality factor can be evaluated from the equivalent series resistance that can be computed from the wire resistance, taking into account the skin depth [49]:

(11) $$R_{a}=\frac{L_{c}}{\sigma \cdot w\cdot \delta (1-exp(-t/\delta ))}$$

where *t* is the thickness of the conductor, σ is its conductivity, and $L_{c}$ is its total length, which for a square shape is

(12) $$L_{c}=4ND_{out}-4Nw-{\left(2N+1\right)}^{2}\left(s+w\right)$$

The skin depth is given by [49]:

(13) $$\delta =\frac{1}{\sqrt{\sigma \mu_{0}\pi f}}$$

Although NFC antennas have a high degree of electromagnetic compatibility with the body compared to other wireless systems that operate at higher frequencies and are based on far-field communication, some challenges must be considered. The reading range is related to the area and quality factor of the antenna. Therefore, in some applications, such as wearable patches, where there are size limitations and integration constraints, the energy-harvesting capacity of the antenna may be limited. Another challenge is the problems associated with the detuning of the system due to the presence of metal parts as happens in smartphones.

To study the influence of the antenna on the reading range, several simulations are performed. Two limit cases are considered. The first is an antenna printed on a typical FR-4 PCB, whose copper plating is 35 μm thick (copper conductivity $5.8\cdot 10^{7}$ S/m). Since the skin depth at 13.56 MHz is less than the strip thickness (17 μm), the designed antennas can have high quality factors. Other substrates, including flexible ones such as polyimide (Kapton), can be used to design PCB antennas. Another traditional technology that can be used to manufacture high quality factor and flexible coils is based on the use of wire coils made with copper. The performance results depend on the thickness of the strip or the diameter of the wire employed. The second case is based on antennas printed with conductive inks [50]. Inks used by the inexpensive Voltera printers are used, with 70 μm thick strips and conductivity of $1.19\cdot 10^{6}$ S/m. Since the skin depth is 125 μm, the antennas have a poor quality factor but offer the advantages of printed electronics, including the choice of substrate, which can be flexible [51]. Another interesting technology for manufacturing NFC antennas is the use of fabrics with conductive threads [52]. In this case, the quality factor of the antennas depends on the conductive thread used [51,53,54].

**Figure 7:**
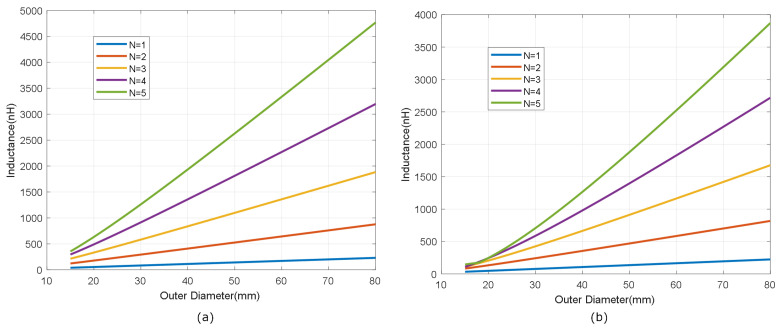
Simulated inductance as a function of outer diameter. (**a**) *w* = 0.5 mm, *s* = 0.5 mm, (**b**) *w* = 1 mm, *s* = 1 mm.

**Figure 8:**
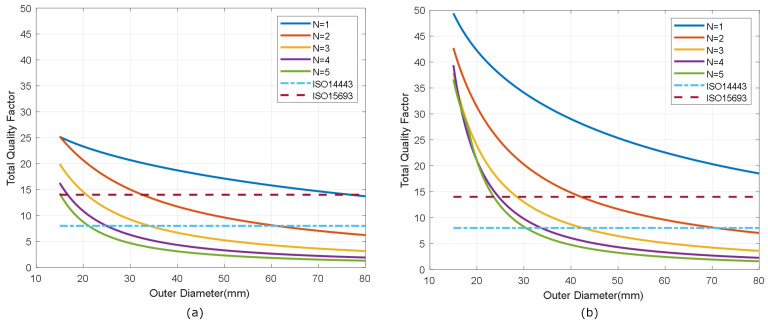
Total tag quality factor for antennas made of copper. (**a**) *w* = 0.5 mm, *s* = 0.5 mm, (**b**) *w* = 1 mm, *s* = 1 mm.

**Figure 9:**
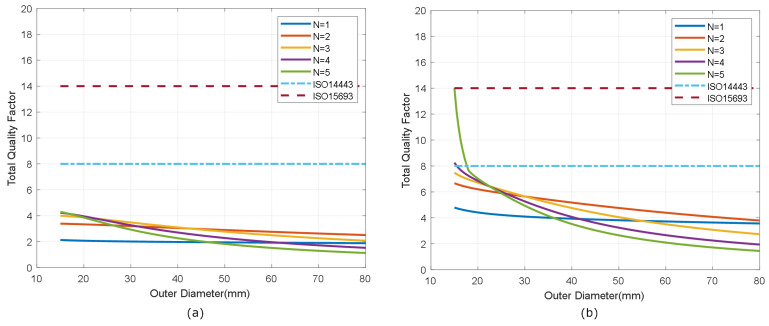
Total tag quality factor for antennas printed with conductive ink. (**a**) *w* = 0.5 mm, *s* = 0.5 mm, (**b**) *w* = 1 mm, *s* = 1 mm.

**Figure 10:**
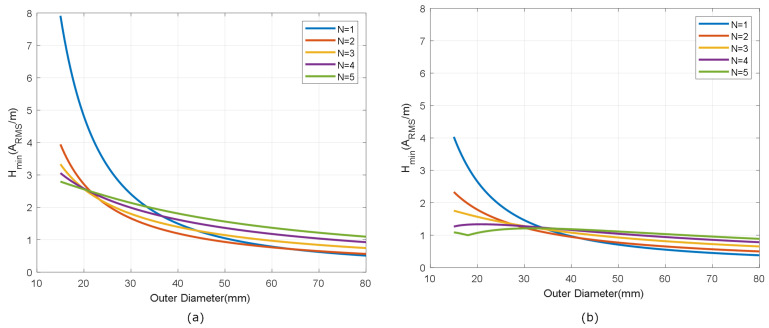
Minimum magnetic field for antennas made of copper. (**a**) *w* = 0.5 mm, *s* = 0.5 mm, (**b**) *w* = 1 mm, *s* = 1 mm.

**Figure 11:**
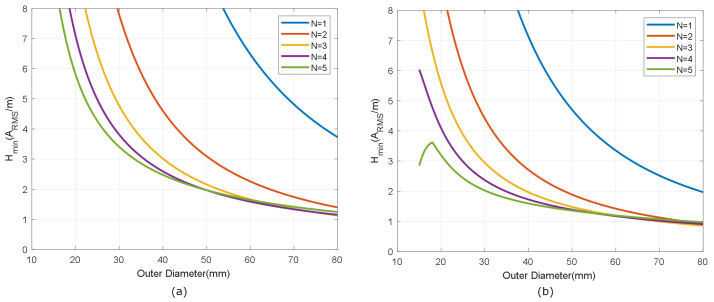
Minimum magnetic field for antennas printed with conductive ink. (**a**) *w* = 0.5 mm, *s* = 0.5 mm, (**b**) *w* = 1 mm, *s* = 1 mm.

**Figure 12:**
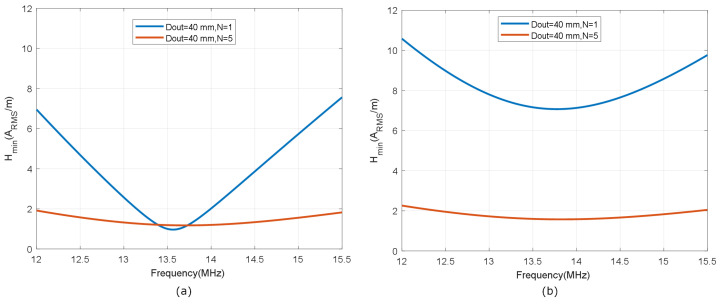
Comparison of minimum magnetic field for an outer diameter of 40 mm and N = 1 and N = 4 turns (*w* = 1 mm, *s* = 1 mm) for (**a**) antennas made of copper, (**b**) antennas printed with conductive ink.

Figure 7 shows the simulated inductance, using Wheeler’s formula, of a square-shaped loop antenna with a different number of turns (N from 1 to 5) for strip widths of *w* = 0.5 mm and *w* = 1 mm. Spacing (*s*) is identical to the width (*s* = *w*). As expected, antennas with smaller widths result in higher values of both inductance and series resistance, and, therefore, lower quality factors. Figure 8 and Figure 9 show the total quality factor of the tag, assuming $R_{IC}=525\;\Omega$, and antennas made of copper or conductive ink, respectively. The maximum quality factors for ISO 1443 and ISO 1593 are also shown. While in the antennas made of copper, the quality factor depends mainly on the IC load, in those printed with conductive ink, the quality factor essentially depends on the antenna. Figure 10 and Figure 11 show the simulated $H_{min}$ at the resonance frequency ($f=f_{r}$), considering an NFC IC with a minimum voltage $V_{min}=4.8V_{RMS}$. The minimum average magnetic field required for energy harvesting increases when the quality factor decreases. In consequence, the reading range of antennas printed with conductive inks is smaller than that of those made of copper. Interesting conclusions are drawn from these figures that can be taken as design guidelines. For high-Q antennas, where the dominant quality factor is that of the IC (outer diameters >25–30 mm), it is preferable to use coils with a small number of turns, contrary to what happens for antennas with poor quality factors, where it is more appropriate to increase the number of turns. Figure 12 plots $H_{min}$ as a function of frequency for a tag with a high-Q antenna (made of copper) and a low-Q antenna (made with conductive ink). If the same outer diameters are considered, the quality factor decreases with the increasing number of turns. Consequently, the bandwidth increases, so the problems associated with detuning (caused for example by the proximity of metals) are less important.

**Figure 13:**
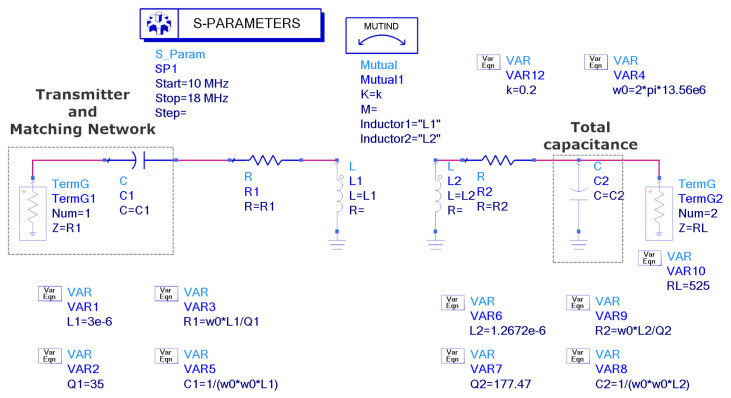
Schema used in Keysight ADS to simulate the reflection coefficient and efficiency.

**Figure 14:**
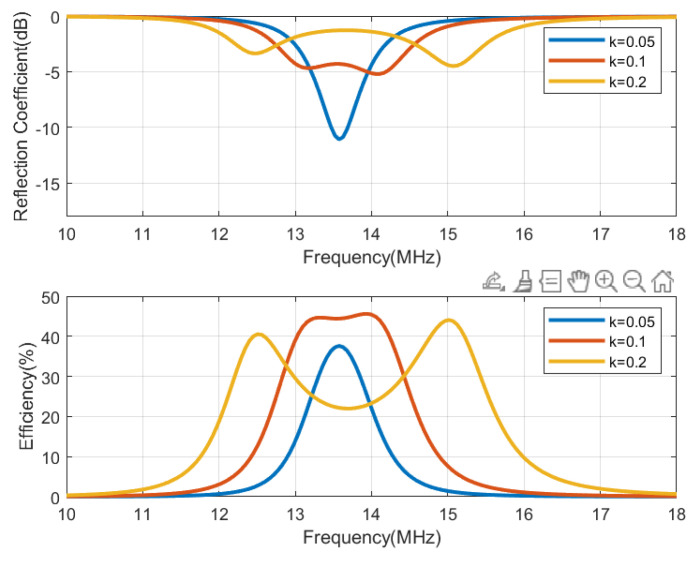
Simulation of the reflection coefficient (**top**) and the efficiency (**bottom**) as a function of the frequency for different coupling coefficients *k* for an antenna made of copper with N = 3 loops.

**Figure 15:**
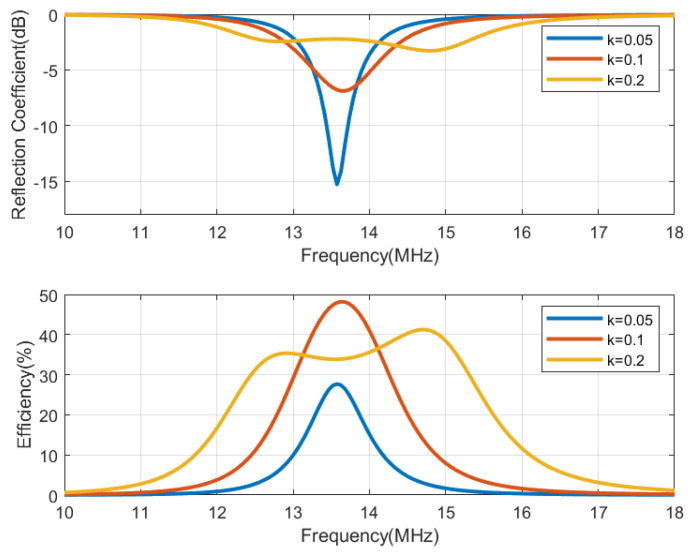
Simulation of the reflection coefficient (**top**) and the efficiency (**bottom**) as a function o he frequency for different coupling coefficients *k* for an antenna made of copper with N = 5 loops.

**Figure 16:**
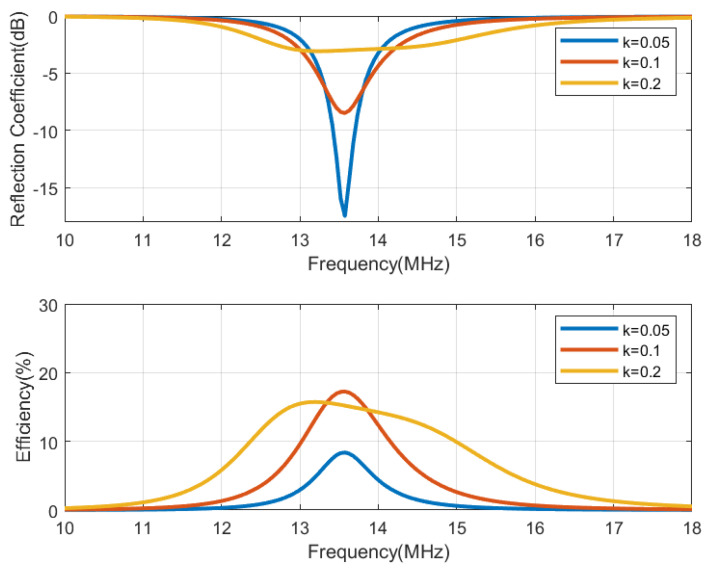
Simulation of the reflection coefficient (**top**) and the efficiency (**bottom**) as a function of the frequency for different coupling coefficients *k* for an antenna printed with conductive ink with N = 3 loops.

**Figure 17:**
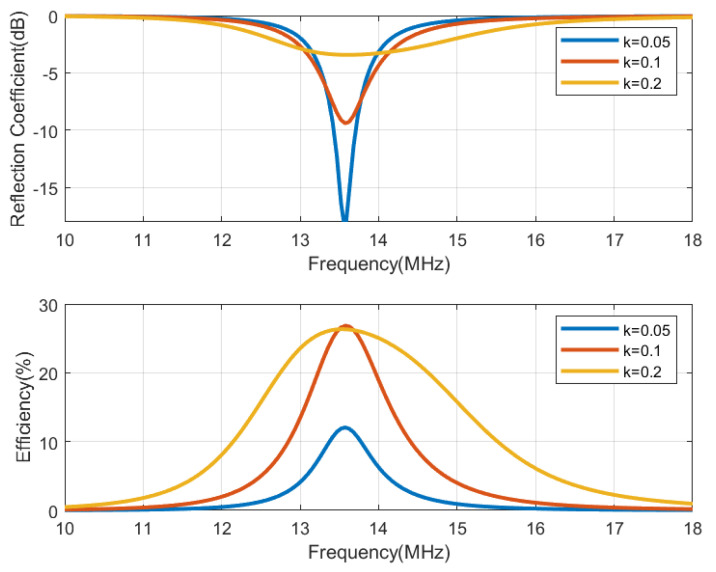
Simulation of the reflection coefficient (**top**) and the efficiency (**bottom**) as a function of the frequency for different coupling coefficients *k* for an antenna printed with conductive ink with N = 5 loops.

The circuit simulation shown in Figure 13, performed with Keysight ADS, is used to generate Figure 14, Figure 15, Figure 16 and Figure 17, which show the reflection coefficient and the efficiency (relationship between the power delivered to the load and the power available from the reader) as a function of the frequency, computed for two antennas, one made of copper and the other printed with conductive ink. Both antennas have an outer diameter of 40 mm, *w* = *s* = 1 mm, and the cases of N = 3 and N = 5 loops are considered. The simulations assume that the transmitter uses an antenna with an inductance of 3 μH, and its quality factor is set to 35 to ensure that it does not limit the bandwidth required for the modulated signal to be transmitted. Both the transmitter and reader are tuned to 13.56 MHz using a matching network in the transmitter and a tuning capacitor next to the tag. The total tag capacitance includes chip and tuning capacitances, as well as parasitic capacitance. The reflection coefficient and efficiency can be calculated by performing an S-parameter simulation, where the reference impedances for ports 1 and 2 are the equivalent resistance of the transmitter and the resistance of the NFC IC, respectively. The reflection coefficient is evaluated from $S_{11}$, and the efficiency is given by ${\left|S_{21}\right|}^{2}$. The NFC IC-equivalent input resistance is considered $R_{IC}$ = 525 Ω. Different coupling coefficients *k* are simulated, which correspond to the cases of weak (when the distance between tag and reader is high, *k* = 0.05), medium (*k* = 0.1), and high coupling (when the distance between tag and reader is small, *k* = 0.2). The mismatch effect on the reader can be observed when the tag is close to the reader. In this case, a reduction in both the efficiency and power received by the tag is observed. This loading effect increases for small tag inductances (N = 3). The use of antennas printed with low-conductivity inks and, therefore, with small quality factors, translates into a noticeable reduction in the efficiency and the harvesting capability.

The previous discussion helps to understand read range limitations and the influence of the tag antenna, but knowledge of two nonlinear parameters of the tag, such as the equivalent IC resistance ($R_{IC}$) and the minimum voltage, $V_{min}$ is also required. These two nonlinear parameters are not provided by the manufacturer. The minimum magnetic field and read range can be obtained experimentally. To this end, the magnetic field generated by the reader must be measured with a test coil antenna (preferably the same antenna that is used in the tag). A procedure to measure the average magnetic field from the power received at the test coil antenna using a spectrum analyzer is described in [31]. To obtain the magnetic field, the antenna factor must be determined from impedance measurements with a VNA. A second, simpler method consists in measuring the RMS voltage using the test coil antenna ($V_{a}$). Since an oscilloscope has a larger input impedance than that of the antenna (typically 1 MΩ in parallel with 15 pF), the average magnetic field can be calculated from Faraday’s law:

(14) $$H_{av}\left(A_{RMS}/m\right)\approx \frac{V_{a}\left(V_{RMS}\right)}{2\pi f_{0}\cdot A\cdot N}$$

where *A* is the area and *N* is the number of turns of the test coil.

**Figure 18:**
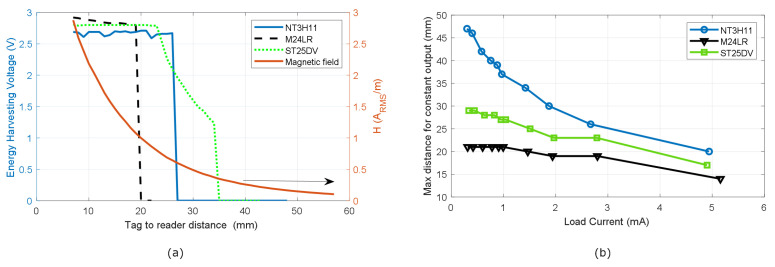
Comparison of reading range for different NFC IC. (**a**) The measured voltage of the energy-harvesting output for the different NFC IC and average magnetic field as a function of the tag to reader distance for a load of 3 mA, (**b**) measured maximum distance for constant voltage at the energy-harvesting output as a function of the load current.

The average magnetic field depends on the reader. It is expected that higher read ranges can be achieved using readers with larger antennas and transmitters with power higher than that of the transceivers integrated on smartphones [33]. As an example, the following figures show read range measurements comparing three commercial NFC ICs with energy-harvesting capabilities [32]. The following NFC ICs are selected: M24LR04E-R and ST25DV from ST Microelectronics, and NT3H11 from NXP. To compare the performance of the different ICs, a coil with a size like that of the smart cards is chosen. Therefore, the read range obtained can be considered representative of general-purpose NFC-based sensors. A 50 × 50 mm loop antenna with 6 turns printed on a 0.8 mm thick FR4 substrate is designed. The width of the strips is w = 0.7 mm, and the gap spacing is s = 1 mm. To investigate the load effect produced by the power consumption of the sensors and the microcontroller, a variable load resistance is connected to the energy-harvesting output of the NFC IC. Figure 18a shows the voltage at the energy-harvesting output as a function of the distance between the tag and the mobile phone used as a reader (Xioami Mi 10 Note 2) for a typical current of 3 mA through the load. The three commercial ICs provide a regulated voltage around 3 V. This figure includes the measured average magnetic field in order to determine the minimum magnetic field ($H_{min}$) necessary for the correct operation of the sensor, which depends on the current in the load. Note that this energy-harvesting range is lower than the read range for reading data previously saved in the NFC IC memory because the ICs do not activate the energy-harvesting output if they do not receive enough power, although they can respond to reader commands. Figure 18b shows the maximum distance reached by the three commercial NFC ICs, where the tag provides a constant voltage on the energy-harvesting output as a function of the current in the load. From these results, it can be concluded that the sensors with current consumption up to 5 mA can be powered by these NFC ICs as long as the magnetic field is high enough. However, in practice, it is advisable to limit it to 3 mA to have a margin faced with the misalignment between coils or the differences in power transmitted between different readers.

## 3. Literature Review of NFC Sensors

**Figure 19:**
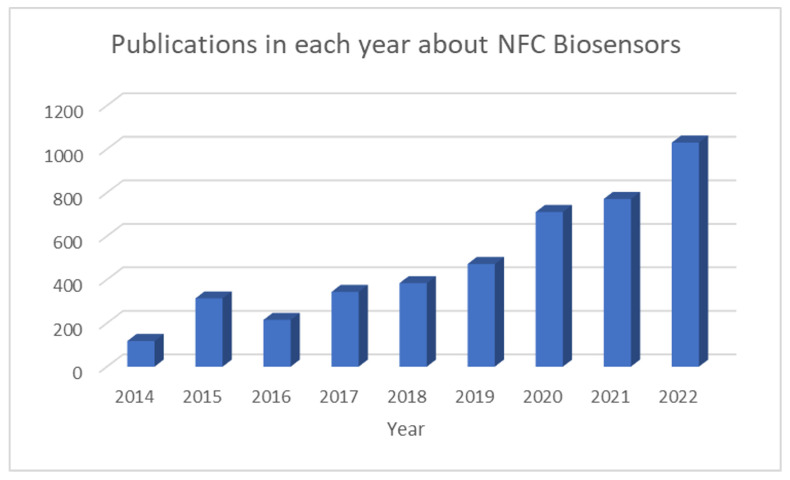
Distribution of the number of publications about NFC biosensors published over the years (source: Dimensions Ai).

As noted above, NFC-based sensors are one of the most promising technologies for the next generation of biosensors. Figure 19 shows the yearly evolution of publications related to NFC biosensors, retrieved from the Dimensions Ai database, which underpins the growing interest in this technology, especially for wearable, implanted, and PoCT devices. This literature review focuses on batteryless devices powered by the energy-harvesting strategies previously discussed, and their applications. The use of batteries is accepted for devices devoted to applications that require high power, such as those to be used for continuous monitoring. In any case, those are beyond the scope of this section. The development of NFC-based sensors involves multiple disciplines that, apart from the communication technologies already discussed, include the use of novel materials to build transducers with the highest sensitivity, and molecular biology to deliver bioreceptors with high affinity towards the target biomarkers [55]. Innovative techniques enabling the access and extraction of bodily fluids stand out, such as microneedle-based technologies [56,57] to reach interstitial fluid, where the concentration of some key biomarkers, such as glucose, is higher and more stable than the levels found in sweat. These new technologies pave the way towards the creation of a new generation of smart patches whose control electronics are integrated into flexible substrates using printing techniques. In addition, thanks to the biocompatibility of the materials used, skin infections can be avoided. Lastly, it is possible to apply machine learning techniques to process the information acquired from different sources.

To discuss the various sensors reported, they are classified according to their transduction method, being colorimetric, electrochemical, capacitive and non-chip sensors. The latter are conceptual but included to provide a broader view.

### 3.1. Colorimetric Sensors

Paper-based microfluidic devices that rely on color changes are a low-cost technique used in various medical applications to support clinical diagnosis, e.g., via pH measurements [58]. These devices are cheap and easy to use, but their accuracy is often limited due to the subjectivity of the user while measuring the color in comparison to a color palette. To overcome this drawback, automatic color measurement using cameras and colorimeters has been proposed [59,60,61,62,63].

Several low-cost batteryless color sensors based on NFC have already been reported as shown in Table 4.

**Figure 20:**
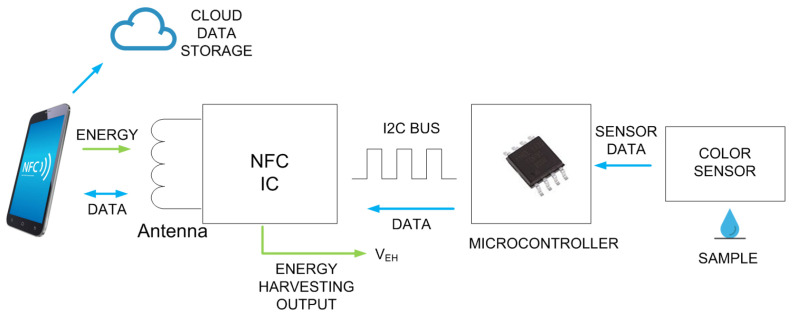
Block diagram of a batteryless NFC-based color sensor.

**Figure 21:**
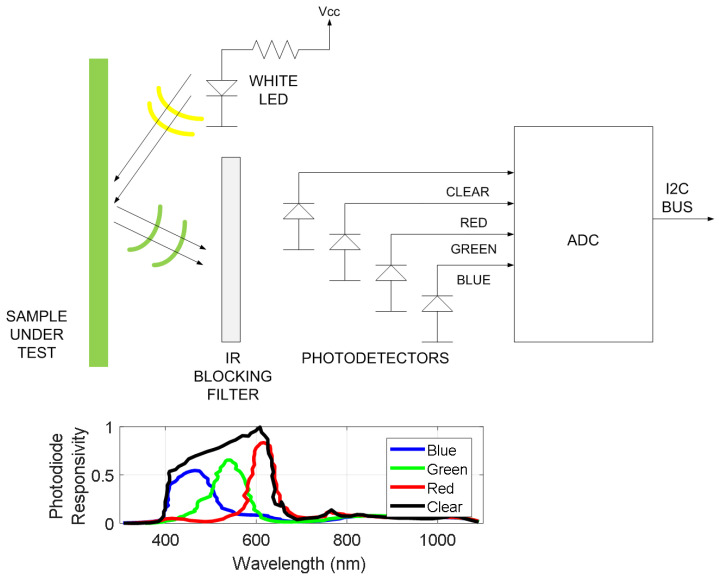
Block diagram of a colorimeter with 4 channels and spectral response of each channel [70].

**Figure 22:**
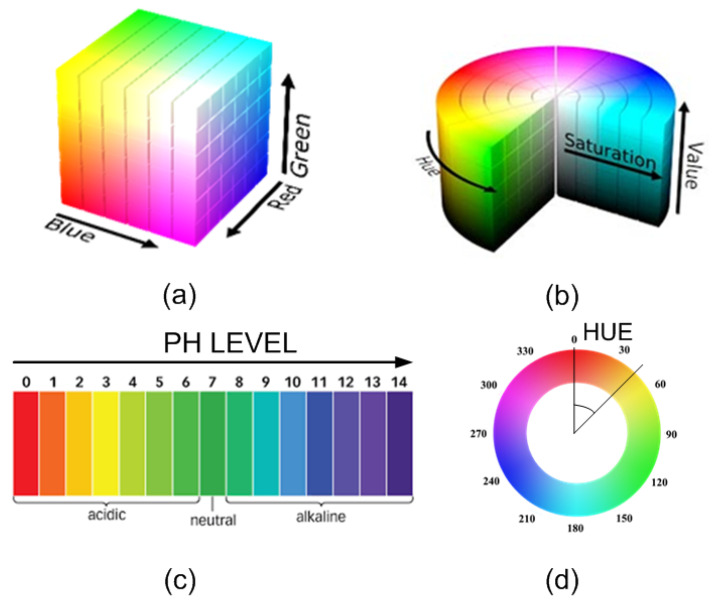
(**a**) RGB color space, (**b**) HSV color space, (**c**) typical color palette of a pH strip, and (**d**) pH as a function of the hue (angle) [70].

**Figure 23:**
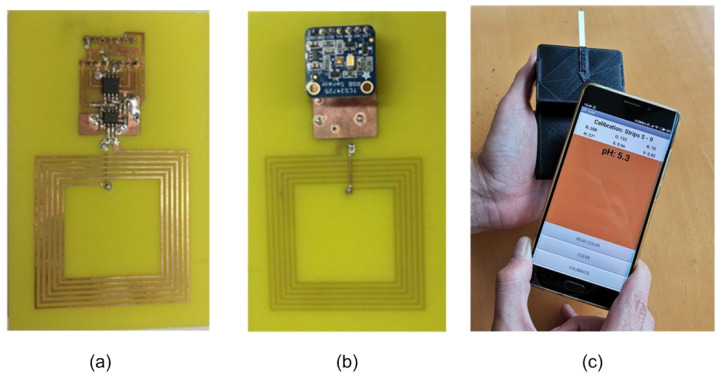
Photograph of a prototype of NFC-based pH sensor: (**a**) top view, (**b**) bottom view and (**c**) reading pH with Android application [70].

**Figure 24:**
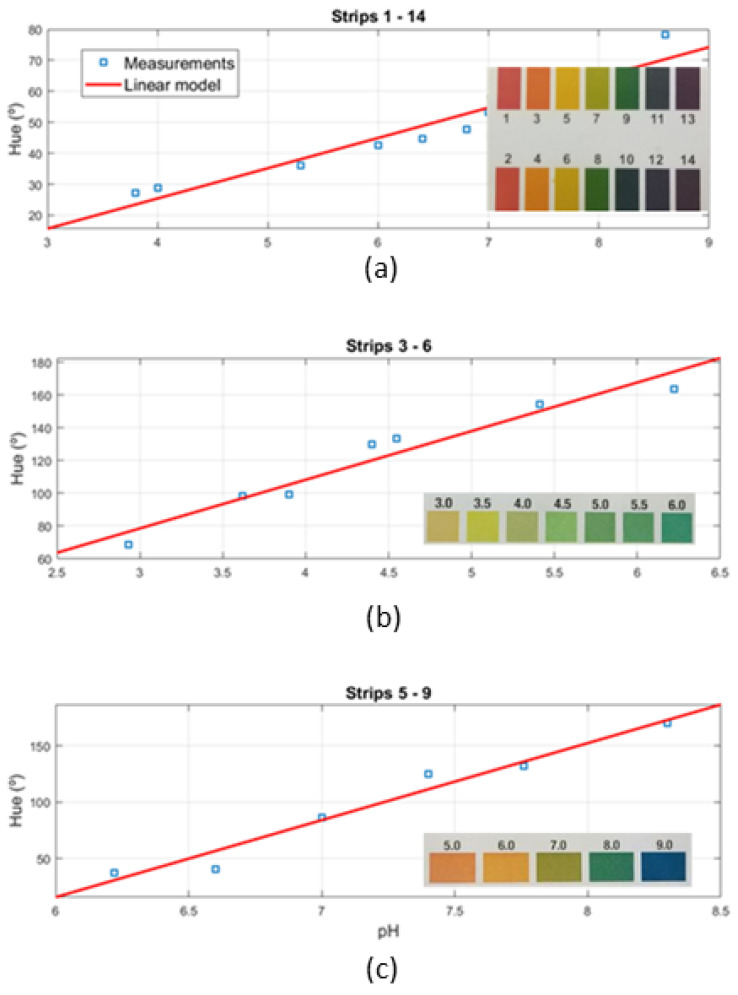
Hue measurements as a function of the pH values for three strips designed to work within different pH ranges: (**a**) strips 1 to 14, (**b**) strips 3 to 6, and (**c**) strips 5 to 9 [70].

The typical block diagram of these color sensors is shown in Figure 20, with the core element being a colorimeter (see Figure 21). These devices consist of an array of photodiodes with a color filter and a single photodiode without this filter (clear channel). As a result, each photodiode is basically sensitive to one color (e.g., red, green, and blue). Photodiodes are covered with an infrared (IR)-blocking filter that minimizes IR spectral components during the color measurement process. Figure 21 also shows the typical spectral response curves for each color channel. The current detected by each photodiode is amplified by a transimpedance amplifier (TIA) and sampled by the analog-to-digital converter (ADC) of a microcontroller. The color data are sent via I2C bus to the NFC IC and after being processed, the results are saved in the EPPROM of the NFC IC in NDEF (NFC data exchange format), compatible with the reader (smartphone). Color measurements using colorimeters are more accurate and repeatable than those made with smartphone cameras since the lighting conditions are controlled [70]. Often, a low-power white LED is used to illuminate the sample under test. The current draw of this LED must be limited due to the NFC IC power restrictions previously discussed. To obtain quantitative results, the discretized RGB measured by the IC colorimeter must be converted to another model or color space. HSV is an alternative representation of RGB, based on how the human eye works. Instead of measuring the portion of each primary color, the results are expressed in terms of hue (see Figure 22b) plotting pure color expressed in degrees from 0º to 360º, saturation (percentage of white color), and value (brightness, expressed in percentage). Figure 23 shows a prototoype of NFC-based pH sensor described in [70] and Figure 24 shows the linear response obtained for hue values measured with the colorimeter integrated into the tag. The method can be extended to other commercial strips to analyze substances such as urea, nitrite, bilirubin, ketone, or glucose in urine, following the same calibration procedure. Using the main concept in [71] a batteryless NFC device was used to classify fruit ripeness, as shown in Figure 23. In this case, the hue and value obtained from the NFC colorimeter were used as input for a classifier algorithm that returned a ripeness level for different fruits (apples, bananas, tomatoes, etc.).

**Figure 25:**
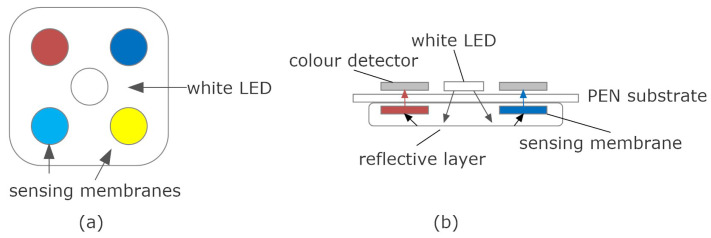
Multi gas sensor based on NFC colorimeter: (**a**) bottom view and (**b**) cross-section. Adapted from [69].

A similar approach was used in [69], using a S11059-02DT (Hamamatsu Photonics, Hamamatsu, Japan) colorimeter to measure color changes of gas-sensing membranes illuminated by a light-emitting diode (LED) situated in the center of the sensing membranes. The tag was made using a flexible and transparent polyethylene naphthalate (PEN) substrate by printing silver conductive ink (see scheme in Figure 25). The membranes were also screen printed. Four sensing membranes with their four-color sensors were used to detect three gases (ammonia, oxygen, and carbon dioxide) and the relative humidity (RH). Sensors responses were provided as color intensity values for the red, green, and blue components measured by a color detector. The tag used an ISL13A NFC IC (ISO15693 standard) and was powered and read from a mobile phone.

In [65], a photometer was implemented using an external optical cell detection system that consisted of red and green LEDs (HSMx-C150, Avago Technologies Ltd., San Jose, CA, USA) and a discrete photodiode (BPW34S, Osram Opto Semiconductors GmbH, Regensburg, Germany). The device was used to quantify the concentration of potassium ions through absorption measurements. The tag used the ISO15693 standard and a low-power microcontroller (PIC12F683 from Microchip Technology Inc., Chandler, AZ, USA). The same authors proposed in [66] a similar system for pH measurements.

**Figure 26:**
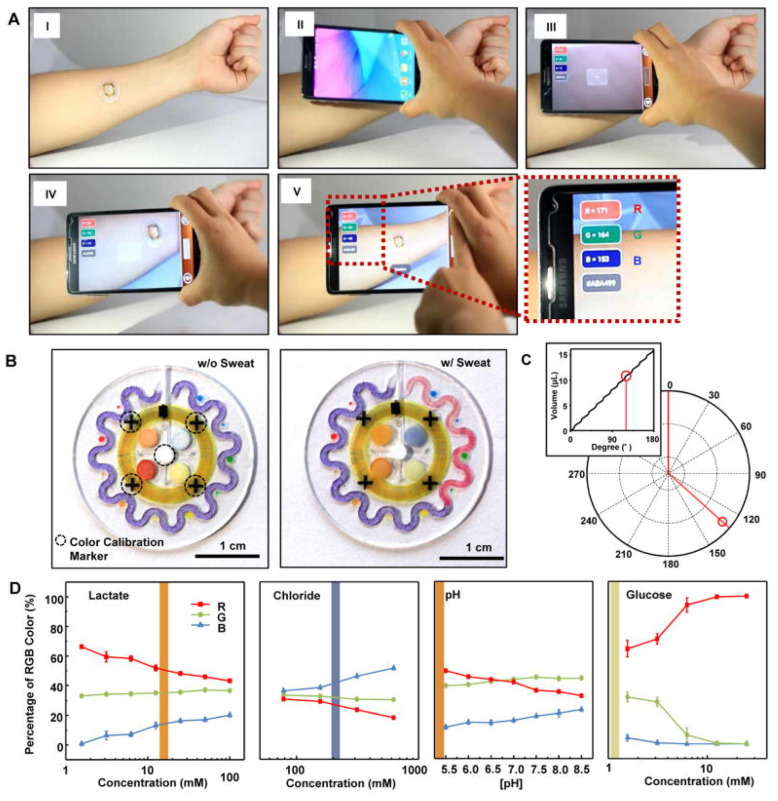
(**A**) Images showing how NFC is used to launch the software for image capture and analysis using the smartphone: (**I**) sensor in the arm, (**II**) sensor focusing with the camera, (**III**) image capture, (**IV**) processing by the smartphone, (**V**) results. (**B**) Images of the epidermal microfluidic biosensor (**left**) before and (**right**) after injecting artificial sweat. (**C**) Location tracking sweat accumulation and their relationship to the total captured volume of sweat (inset). (**D**) Standard calibration curves [67].

In [67], a wearable microfluidic device was reported for sweat analysis. The NFC antenna was mounted on top of an epidermal microfluidic device. NFC communication was used to start the software that allowed capturing and analyzing images, as well as measuring skin temperature. However, the color was measured using the smartphone camera. Quantitative results were provided for sweat rate, total sweat loss, pH, and concentration of both chloride ions and lactate in sweat Figure 26.

In [64], an implantable fluorescent glucose tag based on a custom CMOS ASIC was described. The ASIC integrated the transimpedance amplifier and the AD converter, in addition to managing NFC communication under ISO ISO15693. An antenna printed on a ferrite substrate was used for subcutaneous glucose sensing. The tag dimensions were 3.3 mm in diameter × 15.7 mm in length.

### 3.2. Electrochemical Sensors

Electrochemical sensors are powerful devices for the highly sensitive and real-time monitoring of analytes. Their use is becoming more frequent and increasingly relevant in a wide range of areas and applications, from medical diagnosis to food quality assessment. Regarding diagnosis, electrochemically based PoCT devices that can be used at the patient’s bedside or for the remote monitoring of critical parameters are of great importance in daily clinical routines and emerging e-health applications.

The incorporation of NFC technology within the design of devices based on electrochemical sensors is attracting increasing interest for biomedical applications. These devices can integrate a wide variety of sensors to control temperature, pressure, electrophysiological parameters, blood flow, sweat volume, etc. [72,73]. This section focuses on battery-free electrochemical sensors based on NFC powered by energy harvesting.

Electrochemical sensors rely on the use of electrodes to transduce the interaction of the analyte with the sensing surface producing an electrical signal. Such interactions usually lead to a change in current, potential or conductivity, which gives rise to the so-called amperometric, potentiometric, or conductometric sensors, respectively [74].

Table 5 summarizes some of the battery-free NFC electrochemical sensors reported in the literature.

**Figure 27:**
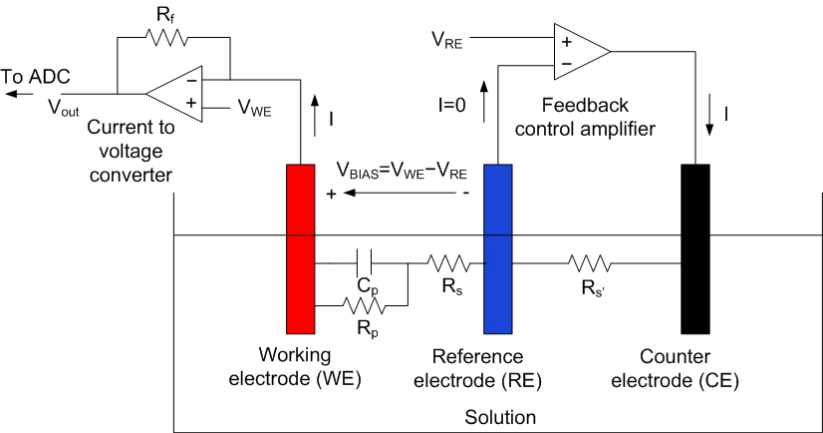
Diagram of a potentiostat with a simplified equivalent circuit for an electrochemical cell.

**Figure 28:**
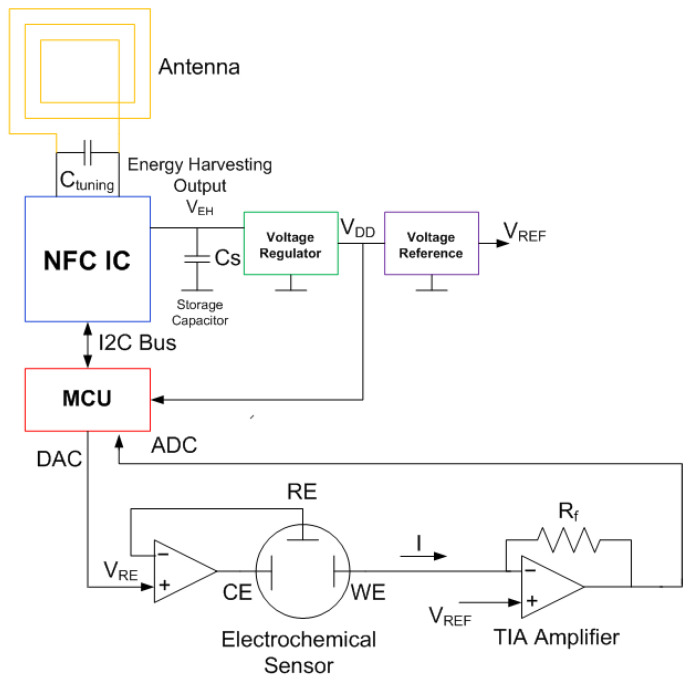
Block diagram of an electrochemical NFC-based sensor.

Amperometric sensors consist of an electrochemical cell with three electrodes (i.e., working/sensing, counter, and reference), and a potentiostat is used as an analog front end (AFE) interface to perform the measurement (see Figure 27). The working principle of these sensors is based on applying a potential to the working electrode that allows the oxidation or reduction of an electroactive species present in the measuring solution, which can be the analyte or is related to the concentration of the analyte to be detected. Therefore, these sensors generate a current, which is directly proportional to the concentration of the analyte. Amperometric biosensors include a bioreceptor (e.g., enzyme and antibody) on the surface of the working electrode that provides specificity towards the target analyte. A transimpedance amplifier is used to measure the low current level. A feedback amplifier is in charge to set the reference potential. In chronoamperometry, the current response to a step bias voltage is measured as a function of time. The end value depends on the resistance $R_{p}$, which, in turn, depends on the concentration. In cyclic voltammetry (CV), the current is measured as a function of the bias voltage. In this case, the peak current depends on the concentration. In both types of measurements, the voltage at the harvesting output of the NFC IC must be stable for the measurement, which can take several seconds. Therefore, a regulated voltage is required. The analog-to-digital conversion can be performed using a microcontroller or, in some cases, using the NFC IC itself. Figure 28 shows the main blocks of an electrochemical NFC-based sensor. In addition to the potentiostat and the transimpedance amplifier, a voltage regulator capable of generating a stable, low-noise reference is essential, besides a microcontroller that has DAC (digital–analog) converters to establish the bias potentials, as well as a low-noise ADC. This scheme allows both chronoamperometry to be performed by biasing the cell with a bias pulse or cyclic voltammetry by sweeping the bias voltage using the DAC. The measured current can be saved in the EEPROM of the NFC IC to be read by the smartphone. The RF field from the reader is used to generate all of the energy. In applications, where continuous monitoring will be required, a battery could be included. The low-power design proposed in this scheme would extend battery life. In certain applications, only the NFC system would be used for communications. The main advantages of amperometric sensors are their high sensitivity, accuracy, and rapid response and recovery time [92].

**Figure 29:**
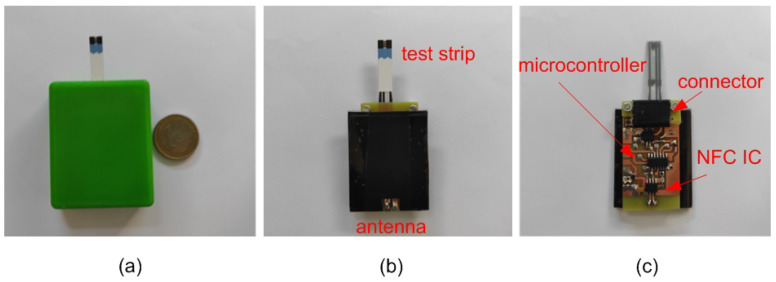
NFC glucometer prototype. (**a**) Prototype including protection case, (**b**) top view, and (**c**) bottom view [76].

**Figure 30:**
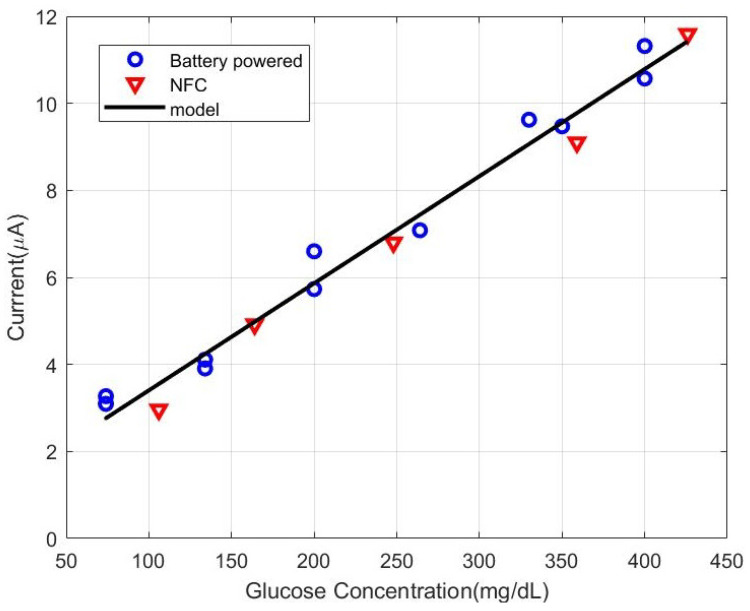
Measured current at 5 s as a function of glucose concentration [76].

In [75], an NFC-based PoC platform based on a commercial potentiostat IC (LMP9001 from Texas Instruments, Dallas, TX, USA) was reported. To increase the dynamic range of the input bias, a voltage doubler circuit was integrated. The sensor also allowed potentiometric measurements using an instrumentation amplifier and a switch, and integrated the NFC IC AS2955 from Austria Micro Systems. In [76], the authors reported a glucose sensor based on commercial glucose strips using a COTS potentiostat consisting of commercial operational amplifiers (see Figure 29). Figure 30 compares the current measured at a sampling instant (after 5 s) of an NFC-based prototype powered by energy harvesting and a commercial battery-powered glucometer. A good agreement between measurements was obtained, and the small differences observed were attributed to the reproducibility of the commercial glucose strips. The NFC-based prototype was used to prepare a calibration curve for the detection of glucose and thus to support its use as a PoCT device. Another example was reported in [80], where an integrated potentiostat manufactured via a standard CMOS process was used to perform cyclic voltammetry measurements. More recently, an integrated NFC chip compatible with ISO14443Aa standard that includes a potentiostat from Silicon Craft Technology PLC (Bangkok, Thailand) was presented in [77] for PoCT applications. The same NFC IC was used to facilitate the amperometric measurements performed with an immunosensor for hepatitis B virus detection in [78] and with a smart-card glucose sensor in [79].

In [45], a wearable patch was used to detect cortisol in sweat through differential pulse voltammetry (DPV). In order to perform the bias voltage sweep at the potentiostat input, a digital-to-analog converter (DAC DAC8562, Texas Instruments Inc., Dallas, TX, USA) was used. The current was measured at the output of the trans-impedance amplifier with an ADC (ADS1115, Texas Instruments Inc., Dallas, TX, USA). Polyethylene glycol terephthalate (PET) was used as a substrate, where the electrodes were screen printed using carbon and Ag/AgCl inks. The working electrode was modified with Au nanoparticles (AuNPs), which were functionalized with thiol-PEG-carboxy to allow the covalent binding of anti-cortisol antibodies to build the finalelectrochemical immunosensor for cortisol detection. Finally, in [83], an ultra-compact glucose tag with a footprint and weight of 1.2 cm${}^{2}$ and 0.13 g, respectively, for sweat analysis was presented.

**Figure 31:**
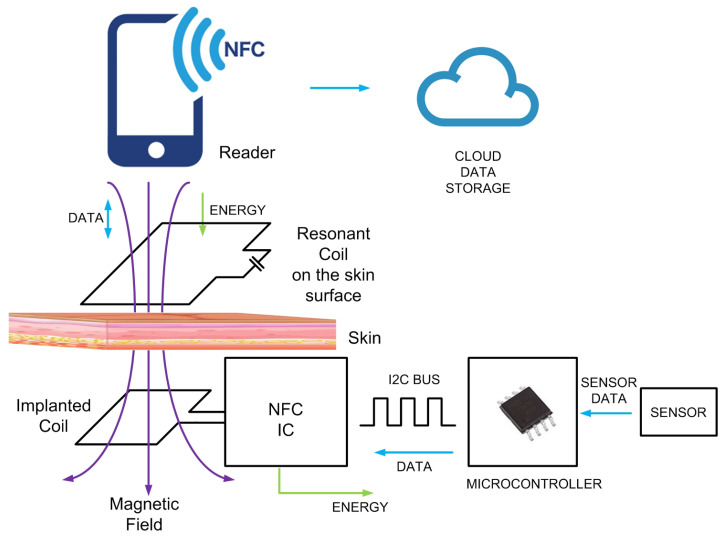
Schema of implanted device with a 3-coil system.

**Figure 32:**
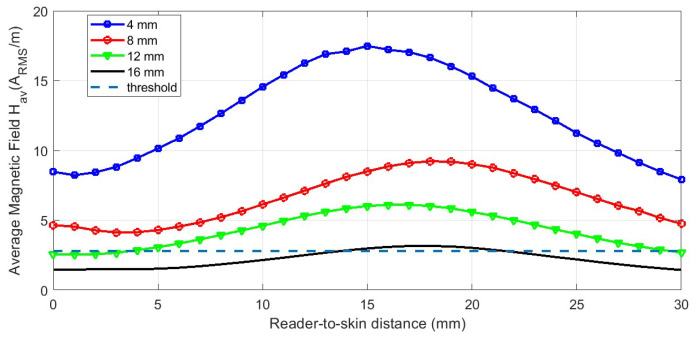
Measured average magnetic field as a function of the reader-to-skin distance for several depths of the tag implanted in the phantom for the 3-coil system. Threshold $H_{min}$ is shown as a dashed line [32].

On the other hand, NFC sensors have great potential for transdermal and implanted devices. The integration of electrochemical sensors has taken these devices to a next level to allow the detection of biomarkers not only on and through the skin but also in deep subcutaneous regions. Information from the transdermal or implanted device can be accessed via a third resonant relay coil, which allows readings with small loop antennas, such as those found in smartphones [32] (see Figure 31). Figure 32 shows the average magnetic field measured as a function of the reader-to-skin distance for different depths of a tag implanted in a phantom gel and using the system with 3 coils (one at the reader, another at the implant, and the relay coil). These results demonstrate that commercial NFC ICs such as ST M24LR04E can be read at depths of 16 mm with the mobile phone placed up to 2 cm from the skin.

Potentiometric sensors measure changes in electrical voltage resulting from the binding of an ion to the ionophore, using suitable bioreceptors and compatible transducers. They typically measure the voltage of either a product’s activity or the activity in an electrochemical reaction within an electrochemical cell, which contains a biological sensing element. Changes in ionic concentrations are determined using ion-selective electrodes. The most used ion-selective electrode is the pH one since many enzymatic reactions involve the release or absorption of hydrogen ions. Besides the pH electrode, ammonia-selective and CO_2_-selective electrodes are also widely spread. However, potentiometric sensors are less sensitive than amperometric ones.

**Figure 33:**
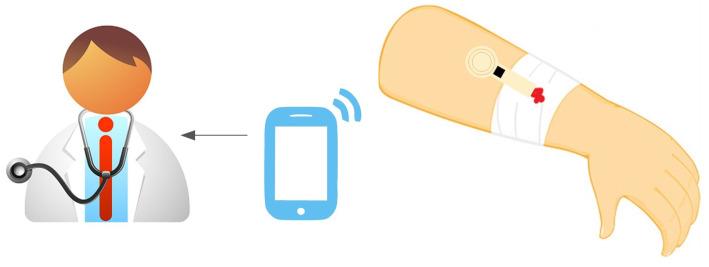
Flexible NFC-based sensing system to monitor pH changes in wounds.

A batteryless glucose sensor based on an abiotic glucose hybrid fuel cell was reported in [85]. The output voltage of the cell was measured with a 14-bit sigma-delta ADC implemented in the NFC IC RF430FRL152H (from Texas Instruments Inc., USA) and stored in the NFC memory to be read from the smartphone. The abiotic glucose fuel cell consisted of an electrode (anode) modified with colloidal platinum (co–Pt), and a cathode made of a composite of silver-oxide nanoparticles and multiwalled carbon nanotubes (Ag2O-MWCNTs). A similar wearable-sensing platform, described in [86], used the same NFC IC to monitor in situ the concentration of [$K^{+}$] in human sweat. The sensor, fabricated on a flexible PET substrate, consisted of a Ag/AgCl reference electrode and a $K^{+}$ selective working electrode made of valinomycin-coated multi-walled carbon nanotubes (MWCNTs). Another example of a potentiometric NFC device was reported in [87], where a disposable pH sensor is presented for wound monitoring applications based on the NFC IC SL13 from AMS (see the schema of Figure 33). The sensor was fabricated by the low-cost direct laser scribing of ITO films.

In [81,82], a batteryless sensor array based on commercial components was developed to potentiometrically detect various ions, including $Na^{+}$, $K^{+}$, and $H^{+}$. The array of potentiometric sensors, printed on a flexible PDMS substrate, was connected to a potentiostat (LMP91002, from Texas Instruments). In [89,90], a proof of concept of a paper-based microfluidic sensor using the SIC 4341 NFC IC 1 (by Silicon Craft Technology) including a potentiostat is shown. The goal is to sense cortisol in sweat. The combination of magnetic beads and paper-based devices opens up a novel approach to sustainable technologies. This innovative concept brings together the advantageous properties of magnetic beads, such as highly efficient antibody immobilization and easy magnet-based manipulation, as well as the various benefits of paper-based devices, including capillary-driven microfluidics that eliminate the need to use external pumps. This fusion of capabilities can be promising in the field of sustainable devices.

Another example using the same SIC 4341 NFC IC was reported in [88], where creatinine (CRE) sensors measured in urine are described. In this case, the commercial screen-printed carbon electrode (SPCE) model CI1703OR from Quasense (Thailand) was used. The working and counter electrodes of the SPCE are coated with carbon, while the reference electrode is coated with silver/silver chloride (Ag/AgCl). A copper oxide (CuO) deposition was obtained for electrodeposition. The formation of the CRE-Cu complex produces an oxidative current, which depends on the creatinine concentrations, and it was measured with a board that integrated the NFC antenna and NFC IC.

In [91], the battery-free NFC electrochemical tag contains a flexible circuit board and an electrode array. The electrode array is composed of two carbon working electrodes, which were modified with AuNPs and BiNPs, respectively, for detections of lead and cadmium that migrated from containers. The board is designed with COST components and implements a square wave anodic stripping voltammetry (SWASV) technique. The tag was fabricated on the polyimide substrate. It includes an NFC chip (NT3H2111, NXP Semiconductor, Eindhoven, the Netherlands), a MCU (MSP430FR2632, Texas Instruments Inc., USA), four operational amplifiers (AD8605, Analog Devices, Wilmington, MA, USA), a 16-bit analog-to-digital converter (ADS1115, Texas Instruments, USA), a 16-bit digital-to-analog converter (DAC8562, Texas Instruments, USA), and an analog multiplexer switch (SN74LVC1G3157, Texas Instruments Inc., USA). The system provides a solution for the in situ analysis of toxic and harmful substances, which can be widely used in the areas of food safety and water quality monitoring.

### 3.3. Capacitive Sensors

**Figure 34:**
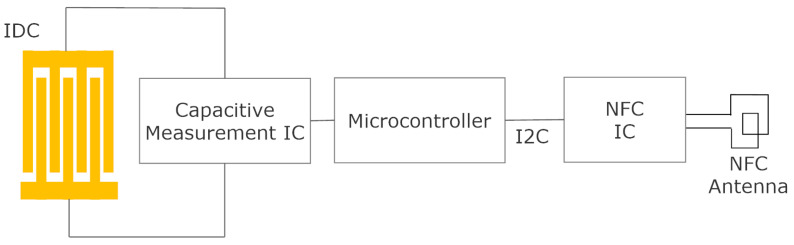
Block diagram of an NFC-based capacitive sensor.

**Figure 35:**
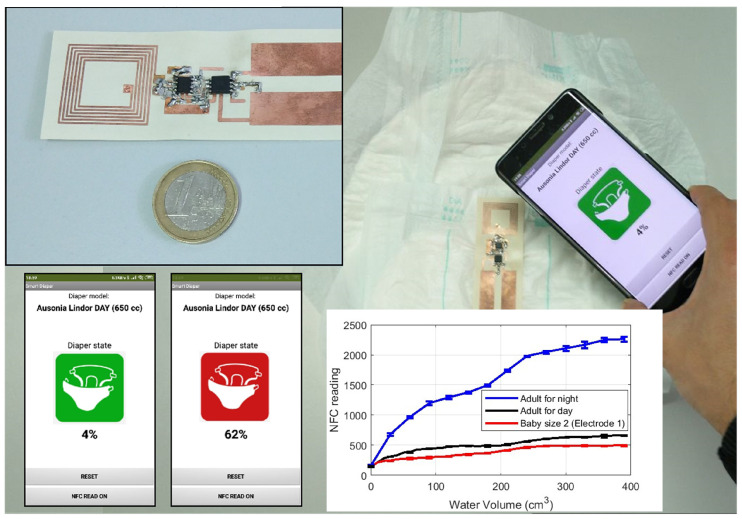
Smart diaper photograph. NFC reading as a function of water volume for different sizes of diapers (Inset) (adapted from [94]).

Capacitive sensors, based on detecting capacitance between two strips or interdigitated electrodes (interdigital capacitor—IDC), can measure biomedical parameters and are compatible with NFC technology (Table 6). Figure 34 shows the typical block diagram of a NFC-based capacitive sensor. Changes in humidity or in chemical composition can vary the relative permittivity, causing changes in capacitance that can be measured and transferred to the mobile phone through NFC. In [93], a capacitive sensor was used to sense changes in the total dissolved solids (TDSs). The capacitance measurement unit was based on a commercial capacitive IC (PCAP02 from AMS AG, Austria), and the sensor was wired and attached to the recipient. The authors of this review proposed a smart diaper based on a capacitive sensor able to detect the presence of urine in the diaper as capacitance changes registered by a microcontroller [94] (Figure 35). Capacitance was obtained from the discharge time of the capacitor measured through a resistor, without the need to add any additional IC for the capacitive sensor. In [95], a battery-free NFC sensor was proposed, consisting of an IDC buried in the soil to measure its humidity. The system relied on a tunable low-pass band RC filter made with the IDC sensor. The variation of the cutoff frequency of the filter allowed detecting capacitance changes thanks to the use of a detector based on diodes connected to the output when the filter was excited with a low-frequency oscillator. The oscillator was based on a low-power 555 timer.

A similar approach was presented in [96], where a skin hydration sensor patch, based on an NFC IDC sensor, was used to predict hydration state. The values recorded by the NFC patch correlated well with those provided by the gold standard reference Corneometer (Courage and Khazaka Company, Cologne, Germany). Ref. [97] reported a device on a wound dressing, combining a commercial humidity and temperature sensor (Sensirion SHT30-ARP) and the NFC IC MLX90129 from Melexis, to control the evolution of wounds. In [98], a humidity detector was developed combining a capacitive IDC, several operational amplifiers and a microcontroller. Capacitance was measured as the difference between a humidity-dependent capacitor and a reference capacitor.

### 3.4. Chipless Sensors

While chipped RFIDs are a well-consolidated technology with a growing market, current research is focused on finding low-cost solutions. One way to reduce the cost of RFIDs is to eliminate the chip. The result is a passive device without a chip, or a chipless RFID. Generally, chipless RFID technology is characterized as a short-range technology. However, this technology may not be competitive in general identification applications due to limitations in bit number coding, although it is interesting for developing low-cost sensors, particularly for wearable devices or low-cost PoCT devices. In these applications, the identification of the sensor can be performed using other approaches if required (e.g., using bar codes, QR codes, or even other RFID chips).

**Figure 36:**
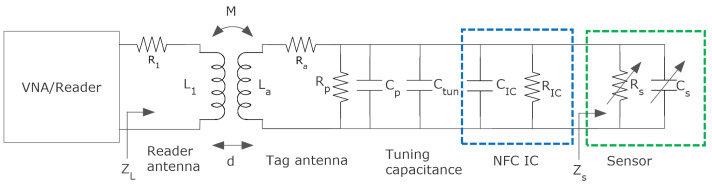
Schema of chipless NFC tag.

In this section, designs based on chipless NFC sensors are reviewed [99,100,101]. These tags are based on the detuning of the response of the antenna. Figure 36 depicts a schema of a near-field chipless tag that is inductively coupled to the interrogating device. The coil antenna is loaded with a sensor that changes its impedance $Z_{s}$ according to the magnitude to measure (e.g., pH and concentration). The equivalent circuit of the sensor can be modeled as a resistance in parallel with a capacitance. A tuning capacitor is added to adjust the resonance frequency at the operation frequency (usually around 13.56 MHz). The change in the sensor capacitance produces a shift in the resonance frequency of the tag, whereas the change in the resistance modifies the quality factor and the amplitude of the response. The reader is a vector network analyzer (VNA), which measures the shift of the resonance frequency and/or amplitude in the measurement of the reflection coefficient. When a qualitative measurement or just detecting the presence of a compound within a certain level is required, an NFC IC for identification can be added to the tag (see blue part in Figure 36). When the sensor detunes the tag, the NFC reader cannot detect it. Therefore, a specialized reader is not required, and a standard NFC reader can be employed [100,101]. In this approach, the tag is not strictly chipless because it integrates an NFC chip, but this IC is used for identification, and from the point of view of the sensor, only its internal capacitance is used to tune the LC resonator. The sensing is passive without the use of any energy harvested from the NFC IC. However, if the operating frequency is on the order of a few MHz and measuring reflection coefficients is required, a low-cost VNA based on SDR (software-defined radio), such as the NanoVNA [102], can be used instead of alternative expensive instruments.

Figure 36 shows the scheme of the operation of chipless NFC technology, showing the input impedance at the reader. The load impedance at the reader near resonance can be written as

(15) $$Z_{L}=R_{1}+j2\pi fL_{1}+\frac{{\left(2\pi f\right)}^{2}M^{2}}{Z_{2}}$$

where $Z_{2}$ is the impedance that loads the tag coil antenna, which is a function of the sensor impedance $Z_{s}$:

(16) $$Z_{2}=R_{a}+j2\pi fL_{a}+\frac{1}{j2\pi f\left(C_{tun}+C_{IC}\right)+1/R_{IC}+1/Z_{s}}$$

The mutual inductance *M* can be expressed as a function of the coupling coefficient *k*, and the distance and misalignment between the two coils:

(17) $$M=k\cdot \sqrt{L_{1}\cdot L_{a}}$$

Therefore, the measurement setup must be the same to calibrate the sensor and measure the tag. To alleviate this drawback and eliminate the self-impedance of the reader coil, the background can be subtracted by previously measuring the impedance in the absence of the tag [103]. After that, the resonance frequency can be obtained from the peak of the real part of $Z_{2}$. Some chipless tags for chemical and biochemical applications are reviewed in Table 7.

In [104], the biosensor consisted of an electrode modified with Ag nanoparticles and glucose dehydrogenase, connected to the antenna modified with a resistive AgCl layer. In the presence of glucose, the enzymatic reaction took place, where glucose was oxidized on the anode, releasing electrons that reduced AgCl to Ag (with high conductivity, reducing the resistance). The AgCl-to-Ag conversion strongly influenced the impedance that loads the antenna and varies the resonance frequency, and the quality factor measured by the VNA.

In [103], a sensor for the detection of basic volatile substances was designed, incorporating a thin layer of hydrogel on a pH-responsive electrode to absorb the volatiles. pH changes caused by the absorbed substances generated a voltage across the pH-sensitive electrode, which changed the capacitance of a varactor (NXP BB202). This varactor loaded the tag coil antenna and produced a change in its resonance frequency. The pH-sensitive electrode consisted of a titanium wire coated by iridium oxide ($IrO_{2}$) and tantalum oxide ($Ta_{2}O_{5}$), while the pH-insensitive reference electrode was made of silver/silver chloride (Ag/AgCl). The sensor was proposed for applications requiring inexpensive and simple detection methods, such as food packaging. Results proved the detection of ammonia ($NH_{3}$).

In [105], a sensor for K+ ions detection was designed, based on an ISE connected to a loop antenna. The sensor showed changes in capacitance as a function of $K^{+}$ concentration, changing the resonance frequency of the tag. Results were obtained from the measurement of parameter S${}_{11}$ when a loop antenna connected to the VNA, 2 cm away from the tag, was used.

In [106] a conductive thin-film polymer (polyaniline—PANi) was chosen to coat the surface of an IDC sensor in an LC resonator to allow ammonia detection. The conductivity of the film increased as a function of the ammonia molecules absorbed on the surface, changing both the amplitude and frequency of the resonance measured with a VNA.

In [107], chipless loop sensors were used to assess milk freshness by applying supervised machine learning. The device used a VNA for the measurement of the reflection coefficient. A coil connected to the VNA was attached to the surface of a milk bottle. The system was based on identifying the most significant frequency components of the data, from the magnitude and phase measurements of the reflection coefficient, to perform a singular value decomposition (SVD). These values were used as features to train a supervised machine learning classifier to detect fresh or spoiled milk. Two frequency ranges were investigated, around 400 MHz and 25 MHz.

The tag coil antenna can be replaced by another type of resonator to increase the operating frequency. A similar concept can be used to design chipless tags based on the change in the resonance frequency of microwave resonators. Resonant antennas can be used in the far-field region to increase the read distance by measuring the variations introduced in the radar cross section of the tag. In [108], a spiral resonator was proposed as a sensor. The activity of a hydrolytic enzyme was measured upon reacting with its specific substrate present within the coating applied on the surface of the resonator. The device measured the degradation rate of the substrate (change in relative permittivity) from the change in resonant frequency. The resonant frequency was determined by measuring the peak of parameter S${}_{21}$ between two antenna coils using a VNA. Another example using a microwave resonator was reported in [109], where a dual-polarized annular slot resonator at 2.4 GHz was used to monitor the moisture content in hermetically packaged food products. In this case, the resonance frequency of the ring resonator was adjusted with a PVA-coated IDC, whose permittivity is sensitive to changes in relative humidity. The authors of this review proposed in [110] a frequency selective surface (FSS) to determine the permittivity of concrete for non-destructive tests. The FSS was composed by capacitively loaded cross-polarizing dipoles, which allowed detecting changes in the permittivity of the wall where the tag was attached. The use of cross-polarizing tags enabled the detection of the tag without the need to apply complex calibration techniques, based on measuring the S${}_{21}$ parameter between the two cross-polarized antennas connected to the VNA. In [111], a microwave sensor for the detection of high-salinity water containing NaCl, KCl, CaCl${}_{2}$, MgCl${}_{2}$, and Na${}_{2}$CO${}_{3}$ was proposed. The design of the sensor combined a microstrip line and a split ring resonator (SRR) with a circular hole in the middle. Parameter S${}_{11}$ was measured with a VNA from 0.5 to 2.2 GHz. Combining the variation of all resonance frequency peaks with principal component analysis (PCA) allowed determining the concentration and the type of salt dissolved in a tube placed over the ring resonator.

## 4. Discussion

This section compares the different sensor technologies previously analyzed according to their principle of operation (colorimetric, electrochemical, capacitive, and chipless) depending on various criteria, such as performance, power consumption, cost, and other considerations (see Table 8).

Chipless sensors have the advantage of low cost, as they do not require components such as additional integrated circuits that increase the cost of the tag. However, their readability can be influenced by environmental factors such as parasitic capacitances that are not considered in the calibration of the LC resonator, changes in the tag shape due to bending, or variations of the inductive coupling due to variations in the distance or orientation of the antennas between the reader and the tag. In addition, they tend to have lower sensitivity and selectivity than other sensors. Therefore, they can be used in applications (e.g., food traceability), where it is essential to know if the parameter of interest exceeds a certain threshold level. In addition, some other important drawbacks are the need to develop a specialized reader to be able to read chipless tags, as well as the lack of standardization.

The rest of the technologies studied in this work use an integrated NFC device, whose mission consists of two objectives: energy harvesting and communications. Therefore, the cost depends mainly on the number of additional components required and the specific sensor technology. The use of a standardized and expanding technology facilitates the reduction in tag costs and the development of mobile applications and specific cloud services for data analysis. In addition, in some biomedical parameter sensing applications with very large potential markets, the analog front end can be embedded in special-purpose ICs, greatly reducing the final cost of the tag.

However, there are particularities in the different technologies studied. In the case of the colorimeter-based sensors, it is necessary to illuminate the sensor with one or more LEDs which significantly increases the power consumption of the tag. They also require a photodetector. These optical devices, apart from increasing the total cost of the tag, are difficult to integrate into the IC together with the microcontroller or other CMOS elements, such as transimpedance amplifiers. The sensitivity of such sensors and the minimum detectable level are generally lower than those of electrochemical sensors. The increasing appearance of NFC-integrated circuits integrating the potentiostat facilitates the commercialization of electrochemical sensors in high-volume applications (e.g., glucose sensors for diabetes monitoring).

Capacitive sensors are mainly focused on applications related to humidity sensors made with some capacitive element. From this point of view, the range of applications compared to that of electrochemical sensors is smaller. Depending on the area of these sensors, the capacitance is enough to use touch sensors integrated into MCU to perform capacitive measurements. This fact reduces the number of components required and power consumption. As in the case of electrochemical sensors, high integrated solutions can reduce the tag cost. The sensitivity of capacitive sensors depends not only on the accuracy of the capacitance measurement but also on the parasitic capacitances of the environment in which the sensor is installed.

## 5. Conclusions and Future Perspectives

NFC technology has been massively established into payment applications. Therefore, most smartphones nowadays include an NFC reader. However, there are other applications that can exploit the wireless communication capabilities of this technology and its ability to harvest energy from the radio frequency signal. NFC technology is showing an increasing potential for IoT applications, as it facilitates the development of wireless sensors without batteries, avoiding environmental problems related to the waste management of batteries. Battery-free designs also allow reducing the number of elements traditionally related to managing their recharge or replacement when discharged. On the other hand, the reading procedures used in NFC systems avoid using electronic displays and, thus, their added cost to the PoCT. Therefore, batteryless NFC sensors can considerably reduce their cost and are particularly interesting in applications where they must be disposed of, such as biomedical, where it is usually mandatory to avoid sample contamination. Powerful mobile-based applications can develop fast from the standardized data interface exchange based on the NDEF format. The reading distance of passive NFC tags is limited to a few centimeters due to the attenuation suffered by the magnetic field as a function of the distance. This fact endows NFC technology with intrinsic security and provides users with a certain level of confidence when used regularly to make payments. The fact that the user’s smartphone is used as a reader provides an additional point of security or privacy. Compared to other RFID technologies, such as UHF RFID, although the read range is smaller, no specific readers are required, and the energy that can be collected is higher. In NFC technology, communication occurs by inductive coupling; therefore, the antennas used are coils and practically do not experience detuning in the presence of high-permittivity materials, such as the human body. Therefore, they can be integrated into skin patches or even implants. For these reasons, NFC wireless sensor technology is expected to become a key technology in future PoCT devices. As a result, those PoCT devices would be cost effective and easy to use, the latter being an essential requirement for both the elderly and future remote monitoring applications.

This review describes the design of energy-harvesting NFC sensors and discusses some examples of recent sensors and biosensors using this technology for various applications (e.g., biomedical). First, it provides an intuitive review of energy harvesting in NFC, which is the key feature to power sensors in tags without batteries. Several important parameters, such as the influence of the size of the coils and their number of turns, as well as the effect of the conductivity of the conductors, are analyzed. Two extreme cases are thoroughly studied to show the degradation caused by using coils printed with low-conductivity inks. In applications that require flexible devices (e.g., wearable devices), depending on the substrate used, a hybrid technology can be used, where coils and electronic circuits are manufactured by conventional techniques (e.g., chemical etching), and the electrodes are printed with conductive inks and functionalized, taking advantage of the inherent advantages of printing techniques. In this way, it is possible to obtain inductors with high-quality factors, improving energy harvesting and facilitating the assembly of the electronic components.

Sensors based on near-field chipless technology are also discussed in the review. These devices can potentially be cheaper than those based on chips, although they have some limitations. The main drawback is the lack of readers on the market, as it is not a standardized technology. However, if frequencies of a few MHz are used, they could be implemented with low-cost software-defined radio (SDR) devices. These sensors may be of interest in some industrial applications.

Within the internet of medical things (IoMT) paradigm, the next generation of connected PoCT devices is a key component. IoMT devices are designed to connect to the internet or local networks, allowing them to transmit data in real time. This connectivity enables seamless data exchange between medical devices, healthcare providers, and cloud-based platforms. IoMT devices continuously collect health-related data from patients, individuals, or medical procedures. These data points can include vital signs, activity levels, blood glucose levels, heart rate, medication adherence or other biochemical parameters. The collected data will be sent to cloud-based platforms or edge computing systems, where sophisticated algorithms and artificial intelligence (AI) processes will analyze the data to generate valuable insights. These insights can be used for personalized healthcare, the early detection of health issues, and the optimization of treatment plans. IoMT enables remote patient monitoring, allowing healthcare professionals to monitor patients’ conditions from a distance. This can lead to better patient outcomes, reduced hospital visits, and improved management of chronic conditions. Challenges of the IoMT paradigm include data security concerns, the interoperability of diverse devices and systems, regulatory compliance, and addressing the digital divide to ensure equitable access to IoMT technologies. The adoption of NFC standards for reading the simplest PoCT and smart patches allows a secure and private method of communication, where the mobile is used as an interface to the future IoMT cloud services. As an additional benefit, the next generation of IomT puts more health-related information in the hands of patients, allowing them to take a proactive role in managing their health and making informed decisions.

## Figures and Tables

**Table 1:** Energy-harvesting sources overview.

Source	Energy-Harvesting Power Density	Drawbacks
Ambient radio frequency	<1 μW/cm${}^{2}$	Distance dependence, useful when in close proximity to a radio transmitter
Ambient light	Directed toward bright sun: 100 mW/cm${}^{2}$	Dependent on indoor lighting. Efficiency depends on the cell technology, typically <20% for outdoors and 1–7% under indoor illumination
	In illuminated office: 100 μW/cm${}^{2}$	
Thermoelectric	60 μW/cm${}^{2}$	Assuming temperature difference of 5K. It requires good thermal insulation of the thermal cell faces.
Vibrational microgenerators	4 μW/cm${}^{3}$ (human motion—Hz)	Highly dependent on the frequency of excitation. Difficult interrogation in wearables except in shoes
	800 μW/cm${}^{3}$ (machines— kHz)	

**Table 2:** Comparison of commercial NFC IC with energy harvesting.

IC	Standard	Energy Harvesting	Comments
ST M24LR-E	ISO 15693	6 mA/3 V	I2C bus
ST ST25DV	ISO 15693	6 mA/3 V	I2C bus
NXP NT3H2111	ISO 14443-3	6 mA/3 V	I2C bus, Regulated output
NXP NTP53221	ISO 15693	6 mA/3 V	I2C, AES authentication, PWM output
TI RF430FRL152H	ISO 15693	NA/3 V	MSP430 MCU integrated
On Semiconductor N24RF64E	ISO 15693	3 mA/3 V at 3.5 A/m	I2C Bus
EM Microelectronic NF4	ISO 14443-A	2 mA	$H_{min}$ = 0.7 A/m, SPI, Tamper detection
AMS AS3955	ISO 14443A	5 mA/4.5 V	SPI/I2C Bus, Regulated output
AMS AS39513	ISO 15693	3 mA/3 V	On-chip temperature sensor and analog input for external resistive sensor, SPI bus
Silicon Craft SIC4340	ISO 14443A	NA	Adjustable current source for resistive and capacitive measurement
Silicon Craft SIC4341	ISO 14443A	NA	Built in potentiostat with current input ranges of 2.5 μA and 20 μA, and bias voltage range from −0.8 V to 0.8 V

**Table 3:** Coefficients for modified Wheeler inductance expression.

Shape	$K_{1}$	$K_{2}$
Square	2.34	2.75
Hexagonal	2.33	3.82
Octagonal	2.25	3.55

**Table 4:** Summary of colorimetric NFC-based sensors reported in the literature.

Reference	Applicattion	NFC IC	Comments
DeHennis 2015 [64]	Implantable glucose sensor	Custom CMOS compatible with ISO15693	Integrated AFE for fluorescent glucose sensor. Implantable with ferrite antenna
Steinberg 2014 [65], Steinberg 2009 [66]	Photometry and pH	N.A. ISO15693	Microchip PIC12F683 MCU. Absorption measurements using a discrete photodiode and green and red LEDs
Koh 2016 [67]	Sweat analysis	AS39513	NFC tag launches camera and temperature measurement
Bandodkar 2019 [68]	Sweat analysis	TI RF430FRL152H	Biofuel cell–based lactate and glucose
Escobedo 2017 [69]	Gas sensor	IDS SL13A	Color sensing membranes
Boada 2019 [70]	pH measurement	M24LR	Hue decomposition to determine pH from paper strip
Lazaro 2019 [71]	Food classification	M24LR	Hue decomposition and classifiers

**Table 5:** Summary of batteryless NFC electrochemical sensors reported in the literature.

Reference	Application	NFC IC	Comments
Escobedo 2019 [75]	Point-of-care	NFC AS3955 and Potentiostat LMP91000, INA321	Amperometry, voltammetry and potentiometry measurements
Lazaro 2022 [76]	Point-of-care	SIC4341	Integrated NFC with potentiostat
Krorakai 2021 [77], Teengam 2021 [78], Promsuwan 2023 [79]	General purpose potentiostat	SIC4341	Integrated NFC with potentiostat
Boada 2021 [32]	Deep implanted devices	ST M24LR04E	Relay resonant loop
Siegl 2018 [80]	Cyclic voltammetry (CV)	Integrated CMOS with NFC	Potentiostat validated with dummy devices
Xu 2019 [81,82]	Sweat monitoring	NT3H2111	MSP430FR5959, LMP91002 potentiostat, AD8608 amplifiers from Analog Device.
Mirzajani 2022 [83]	Sweat glucose sensor	NA	Amperometry
Cheng 2021 [84]	Analysis of sweat cortisol	NT3H2111	Differential pulse voltammetry with a potentostat using an DAC DC8562 and AD AD1115
Banerjee 2022 [85]	Wearable glucose sensor	RF430FRL152H	Measurement of an abiotic glucose fuel cell
Zhang 2021 [86]	Wearable sweat [$K^{+}$] sensor	F430FRL152H	Ag/Cl and MWCNTs coated with valinomycin
Rahimi 2017 [87]	pH wound monitoring	SL13	direct laser scribing of ITO film
Hongboontry 2021 [88]	Creatinine sensor in urine	SIC 4341	Screen-printed carbon electrodes with CuO film deposited
Bianca 2022 [89], Fiore 2023 [90]	Cortisol analysis in sweat	SIC 4341	Paper-based mcicrofluidic device
Xu 2020 [91]	In situ monitoring of heavy metals	NT3H2111	Square wave anodic stripping voltammetry (SWASV) technique

**Table 6:** Summary of batteryless NFC capacitive sensors reported in the literature.

Reference	Applicattion	NFC IC	Comments
Qian 2021 [93]	TDS	M24LR16E	Measurement of capacitance performed by PCAP02
Lazaro 2019 [94]	Smart diaper	M24LR04E and ATTiny85	Measurement of the capacitance is performed by the microcontoller
Boada 2018 [95]	Soil Moisture	M24LR04E and ATTiny85	Tunable low-pass filter and a diode detector. Integrate a temperature and humidity sensor.
Flament 2021 [96]	Dermatology, Skin moisture sensor	Microcontroller with NFC	External capacitive sensor connected to the microcontroller. In vivo measurements in different body positions.
Li 2020 [97]	Wound bandage monitoring	Melexis MLX90129	Measurement of humidity and temperature with a commercial sensor Sensirion SHT30-ARP
Eldebiky 2018 [98]	Capacitive Humiditiy sensor	EM NF4 and EM6819 microcontroller	Measurement of capacitance using charge amplifier

**Table 7:** Summary of passive near-field sensors reported in the literature.

Reference	Applicattion	Technique	Comments
Shafaat 2022 [104]	Glucose sensor	Inductive coupling at 13.5–18 MHz	Redox reaction-to-resistance transducer coupled to an RF antenna
Bhadra 2014 [103]	Basic volatile sensor (ammonia)	Inductive coupling at 6 MHz	Hydrogel on a pH electrode to control a varactor
Wu 2021 [105]	Potassium ion sensing	Inductive coupling at 14.5 MHz	Integrated antenna and ISE electrode on Kapton substrate
Karuppuswami 2020 [106]	Digital ammonia sensor for packaged food	Inductive coupling at 2.87 MHz	Capacitor coated with a conductive polymer (PANi) as spoilage indicator
Rodriguez 2021 [107]	Beverage freshness detection	Coils at 25 MHz and 400 MHz	Supervised learning machine technique applied to amplitude and phase of measured reflection coefficients
Charkhabi 2018 [108]	Hydrolytic enzyme activity	Spiral resonator at 75 MHz	Measurement of frequency shift of the resonator from S21
Raju 2021 [109]	Relative humidity	Slot annular resonator at 2.4 GHz	PVA-coated IDC
Lazaro 2018 [110]	Structural health testing	Depolarizing FSS	Permittivity of the wall modifies the capacitance and the resonance frequency of the dipoles
Harnsoongnoen 2021 [111]	Dissolved salt determination	Split ring resonator	Frequency shift of multiple resonance, and PCA analysis for classification

**Table 8:** Comparison of batteryless NFC biosensors.

Measurement Principle	Performance	Power Consumption	Cost	Comments
Colorimetric	Medium	High	Medium	Can require sensible strips
Electrochemical	Best	Medum	Medium	Potential low-cost using integrated potentiostats
Capacitive	Medium	Low	Medium	Potential low-cost using integrated capacitive sensors in MCU. Sensible to parasitic capacitances.
Chipless	Low	None	Low	Suitable for threshold applications, Measurement sensitive to coupling. No commercial reader is available and lack of standards

## Data Availability

The data presented in this study are available on request from the corresponding author.
